# Summary Intervals for Model-Based Classification Accuracy and Consistency Indices

**DOI:** 10.1177/00131644221092347

**Published:** 2022-04-28

**Authors:** Oscar Gonzalez

**Affiliations:** 1 The University of North Carolina at Chapel Hill, USA

**Keywords:** classification accuracy, classification consistency, confidence intervals, factor model, screening

## Abstract

When scores are used to make decisions about respondents, it is of interest to estimate classification accuracy (CA), the probability of making a correct decision, and classification consistency (CC), the probability of making the same decision across two parallel administrations of the measure. Model-based estimates of CA and CC computed from the linear factor model have been recently proposed, but parameter uncertainty of the CA and CC indices has not been investigated. This article demonstrates how to estimate percentile bootstrap confidence intervals and Bayesian credible intervals for CA and CC indices, which have the added benefit of incorporating the sampling variability of the parameters of the linear factor model to summary intervals. Results from a small simulation study suggest that percentile bootstrap confidence intervals have appropriate confidence interval coverage, although displaying a small negative bias. However, Bayesian credible intervals with diffused priors have poor interval coverage, but their coverage improves once empirical, weakly informative priors are used. The procedures are illustrated by estimating CA and CC indices from a measure used to identify individuals low on mindfulness for a hypothetical intervention, and R code is provided to facilitate the implementation of the procedures.

Tests scores and scales scores are often used to make decisions about individuals. Examples include using scores to determine if a student meets a minimal level of proficiency (Lee, 2010), if a respondent is flagged by a screener for further treatment (Gonzalez et al., 2021), or if an individual should obtain a license to practice a profession (Moses & Kim, 2015). In many situations, responses from the test or scale are aggregated into an observed summed score $X_{i}^{*}$ , and then one determines if the summed score is above or below a cutpoint (Youngstrom, 2014). A critical measurement concern when using scores to make decisions is describing their expected classification accuracy (CA) and consistency (CC; American Educational Research Association et al., 2014).

CA refers to the probability of making a correct classification during the decision process. Three indices that describe CA are the classification rate, sensitivity, and specificity (Pepe, 2003; Youngstrom, 2014). The classification rate refers to the probability of making a correct decision. Sensitivity refers to the probability of correctly classifying individuals who meet the condition (e.g., identify students who are truly proficient or select respondents who truly need the treatment). Specificity refers to the probability of correctly classifying individuals who do not meet the condition (e.g., identify students who are not proficient or rule out respondents who do not need the treatment). In this article, those three indices are referred to as CA indices. For the CA indices, the true condition is determined by a latent variable score (e.g., scores from a gold standard, a clinical interview, etc.), but in applied settings, the classification of respondents is often based on the summed score $X_{i}^{*}$ , which suffers from measurement error. However, classification consistency refers to the probability of making the same decision across two parallel administrations of the same test or scale (Livingston & Lewis, 1995). Note that consistency differs from accuracy in that consistency does not describe if the classification is correct or not, it just has to be the same. The estimation of classification consistency assumes that there is no change on the construct (e.g., changes due to maturation, carry-over effects, treatment effects, etc.) between the administration of the measures (Gonzalez et al., 2021). Throughout the article, this index is referred to as the CC index.

In situations in which one has respondent-level classifications at Time 1, classifications at Time 2, and true classifications, the estimation of the CA and CC indices is a relatively simple task—one can cross-tabulate classifications at Time 1 with true classifications to estimate the CA indices, and cross-tabulate classifications at Time 1 and Time 2 to estimate the CC index (Gonzalez et al., 2021). In this case, the CA and CC indices are proportions, so uncertainty for CA and CC indices could be estimated using confidence intervals for proportions (Newcombe, 1998). However, limited resources might prevent researchers from having either true classifications or scores from a second administration of the measure. Therefore, model-based estimates of CA and CC that require only one administration of the measure have been recently proposed and investigated (Gonzalez et al., 2021; Lee, 2010; Millsap & Kwok, 2004).

Previous research has shown that, when responses to the test or scale are treated as continuous, CA and CC indices can be calculated from the parameter estimates of a linear factor model (further discussed below; Millsap & Kwok, 2004). However, uncertainty estimation for model-based CA and CC has not been widely discussed (although see Gonzalez et al., 2021, for a brief discussion). Moreover, when the point estimates of factor model parameters are used to derive model-based CA and CC, the factor model parameters are treated as fixed, but these parameters suffer from sampling variability. In practice, this source of uncertainty is ignored (e.g., Gonzalez & Pelham, 2021; Lee, 2010; Millsap & Kwok, 2004), but there is a possibility that it may be consequential to the estimation of summary intervals for CA and CC indices. A promising solution would be to estimate the uncertainty of model-based CA and CC indices using the bootstrap (Efron & Tibshirani, 1993) or Bayesian estimation (Levy & Mislevy, 2017). Both approaches provide natural frameworks to propagate the uncertainty of factor model parameters to derived quantities via their sampling procedures (Kelley & Pornprasertmanit, 2016; Levy & Mislevy, 2017). Recent work suggests that percentile bootstrap confidence intervals (Kelley & Pornprasertmanit, 2016) and Bayesian credible intervals (Pfadt et al., 2021; Tanzer & Harlow, 2021) have appropriate coverage on the estimation of test score reliability. As such, it is expected that the bootstrap and Bayesian summary intervals^1^ could be viable approaches to estimate the uncertainty of CA and CC indices, although this has not been investigated.

This article has three goals: (a) show how to estimate bootstrap confidence intervals and Bayesian credible intervals for model-based CA and CC indices, (b) evaluate if the summary intervals have appropriate interval coverage of the true value, and (c) facilitate the reporting of the uncertainty of model-based CA and CC indices by providing R code. The structure of the article is the following. First, the estimation of CA and consistency using parameters from the linear factor model is introduced. Then, background on bootstrapping and Bayesian inference and how they can be used to incorporate parameter uncertainty to the summary intervals of CA and CC indices is described. Next, the estimation and interpretation of bootstrap and Bayesian summary intervals are shown using an applied example. Finally, results from a small simulation study on the coverage of the percentile bootstrap and Bayesian summary intervals for the CA and CC indices are discussed. In the supplement, R code is provided to assist researchers on the implementation of this methodology.

## Estimating Model-Based CA and CC

Below, some implied relations of the linear factor model for the expected mean and variance of summed scores are introduced, and then their connection to model-based CA and CC indices is reviewed.

### Linear Factor Model and Some Implied Relations

Let $X_{\mathrm{ij}}$ be the score of respondent *i* on item *j*. When $X_{\mathrm{ij}}$ is continuous and the items are homogeneous, the relationship between the $X_{\mathrm{ij}}$ and the latent variable η underlying the assessment can be described using a unidimensional linear factor model,

(1) $$X_{\mathrm{ij}}=\tau_{j}+\lambda_{j}\eta_{i}+\epsilon_{\mathrm{ij}}.$$

In this case, $\tau_{j}$ is the intercept of item *j*, $\lambda_{j}$ is the factor loading for item *j*, and $\epsilon_{\mathrm{ij}}$ is the unique score for person *i* on item *j*, with variance $\psi_{\mathrm{jj}}$ across individuals. Recall that researchers typically make decisions using the summed item responses for each respondent, $X_{i}^{*}$ . Consequently, the relations implied by the linear factor model can be used to calculate the expected mean $\mu_{X^{*}}$ and variance $\sigma_{X^{*}}^{2}$ of $X^{*}$ (Millsap & Kwok, 2004),

(2) $$\mu_{X^{*}}=\tau^{*}+\lambda^{*}\kappa \;\;\;\sigma_{X^{*}}^{2}=\lambda^{{*}^{2}}\Phi +\psi^{*}.$$

In this case, $\tau^{*}$ is the sum of the intercepts, $\lambda^{*}$ is the sum of the factor loadings, $\psi^{*}$ is the sum of the variances of the unique scores, κ is the mean of η, and Φ is the variance of η. For the estimation of the CA indices, we need to calculate $\mathrm{Cor}\left(X^{*},\;\eta \right)$ , the expected correlation between $X^{*}$ and η (Millsap & Kwok, 2004),

(3) $$\begin{array}{c} \mathrm{Cor}\left(X^{*},\;\eta \right)=\frac{\mathrm{Cov}\left(X^{*},\eta \right)}{\sqrt{\mathrm{Var}\left(X^{*}\right)}\sqrt{\mathrm{Var}\left(\eta \right)}}=\frac{\mathrm{Cov}\left(\tau^{*}+\lambda^{*}\eta ,\;\eta \right)}{{\left(\lambda^{*2}\Phi +\psi^{*}\right)}^{1/2}\Phi^{1/2}}\\ =\frac{\lambda^{*}\Phi }{{\left(\lambda^{*2}\Phi +\psi^{*}\right)}^{1/2}\Phi^{1/2}}=\frac{\lambda^{*}\Phi^{1/2}}{{\left(\lambda^{*2}\Phi +\psi^{*}\right)}^{1/2}}, \end{array}$$

which simplifies even further when the linear factor model is identified by setting Φ = 1. For the CC index, we need to calculate $\mathrm{Cor}\left(X_{1}^{*},\;X_{2}^{*}\right)$ , which is the correlation of the summed score for two administrations of the measure in quick succession. Recall that the CC estimation assumes parallel administrations of the measure, which presumes $\mu_{X_{1}^{*}}=\mu_{X_{2}^{*}}=\mu_{X^{*}}$ and $\sigma_{X_{1}^{*}}^{2}=\sigma_{X_{2}^{*}}^{2}=\sigma_{X^{*}}^{2}$ , along with longitudinal invariance. The implied $\mathrm{Cor}\left(X_{1}^{*},\;X_{2}^{*}\right)$ can be estimated using,

(4) $$\mathrm{Cor}\left(X_{1}^{*},\;X_{2}^{*}\right)=\frac{\mathrm{Cov}\left(X_{1}^{*},X_{2}^{*}\right)}{\sqrt{\mathrm{Var}\left(X_{1}^{*}\right)}\sqrt{\mathrm{Var}\left(X_{2}^{*}\right)}}=\frac{\mathrm{Cov}\left(\tau^{*}+\lambda^{*}\eta ,\;\tau^{*}+\lambda^{*}\eta \right)}{{\left(\lambda^{*2}\Phi +\psi^{*}\right)}^{1/2}{\left(\lambda^{*2}\Phi +\psi^{*}\right)}^{1/2}}=\frac{\lambda^{{*}^{2}}\Phi }{\lambda^{*2}\Phi +\psi^{*}},$$

which is the equation for coefficient omega, a commonly used estimator of reliability for homogeneous tests (McDonald, 1999), which is also equivalent to $\mathrm{Cor}{\left(X^{*},\eta \right)}^{2}$ from Equation 3.

### Bivariate Distribution and Indices

**Figure 1.**
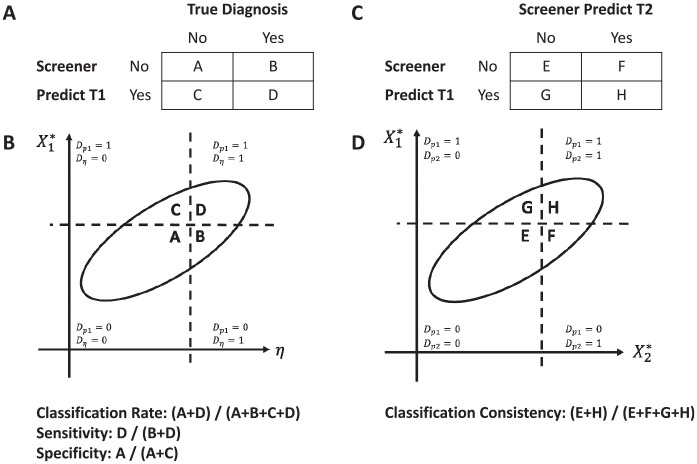
Relation Between the Bivariate Normal Distributions and 2×2 Tables to Estimate Classification Accuracy and Consistency; *Dp*_1_, *Dp*_2_, and *D*_eta_ Indicates the Determined Diagnosis. (A) Panel A is a 2x2 table of the true diagnosis and the predicted diagnosis by the screener, (B) Panel B is the bivariate normal distribution of the latent variable and the screener score at time 1, (C) Panel C is a 2x2 table of the predicted diagnosis at time 1 and time 2, and (D) Panel D is the bivariate normal distribution of the screener score at time 1 and time 2.

Collecting the preceding developments, one can define two bivariate normal distributions (see Figure 1) used in the estimation of CA and CC indices, which map to the decision cross-tabulation described above (see Figure 1A and 1C). In an ideal scenario, one would select individuals who are above a cutpoint on η, which represents a gold standard, but because latent variable scores are not observed and often not used in practice, selection takes place using the observed summed score $X^{*}$ with respect to a cutpoint on $X^{*}$ . To study the relation between selecting individuals based on $X^{*}$ and on η, one can define a bivariate normal distribution based on $\mu_{X^{*}},\;\sigma_{X^{*}}^{2},\kappa ,\;\Phi ,$ and $\mathrm{Cor}\left(X^{*},\;\eta \right)$ and cutpoints on $X^{*}$ and η can be imposed. Imposing the cutpoints on the distribution forms quadrants A, B, C, and D (see Figure 1B), which are defined as follows: C is the proportion of respondents who were above the cutpoint on $X^{*}$ but below the cutpoint on η (a false positive; an incorrect classification), D is the proportion of respondents who were above both the cutpoint on $X^{*}$ and the cutpoint on η (a true positive; a correct classification), A is the proportion of respondents who were below both the cutpoint on $X^{*}$ and the cutpoint on η (a true negative; a correct classification), and B is the proportion of respondents who were below the cutpoint on $X^{*}$ but above the cutpoint on η (a false negative; and incorrect classification). The proportion of the bivariate normal distribution in each quadrant, estimated via integration, is then used to estimate the CA indices. One could estimate classification rate, the probability of making a correct classification, as (A+D)/(A+B+C+D), sensitivity, the probability of correctly classifying those who meet the condition determined by η, as D/(B+D), and specificity, the probability of correctly classifying those who meet the condition determined by η, as A/(A+C). Similarly, one could estimate the CC index by imposing the cutpoints on $X^{*}$ on the bivariate normal distribution based on $ $\mu_{X_{1}^{*}},\mu_{X_{2}^{*}},\sigma_{X_{1}^{*}},\sigma_{X_{2}^{*}},$ and $\mathrm{Cor}\left(X_{1}^{*},\;X_{2}^{*}\right)$ . Recall that parallel tests have the same mean and variance, so $\mu_{X_{1}^{*}}=\mu_{X_{2}^{*}}=\mu_{X^{*}}$ and $\sigma_{X_{1}^{*}}^{2}=\sigma_{X_{2}^{*}}^{2}=\sigma_{X^{*}}^{2}$ , and the cutpoint on $X_{1}^{*}$ is the same as the cutpoint on $X_{2}^{*}$ . Imposing the cutpoints on the distribution forms quadrants E, F, G, and H, shown in Figure 1D, and defined similar to quadrants A, B, C, and D in Figure 1B. Classification consistency, the probability of making the same classification in two parallel administrations of the measure, can be estimated as (E+H)/(E+F+G+H).

Note that on the estimation of the CA and CC indices, we need to determine $\mu_{X^{*}}$ , $\sigma_{X^{*}}^{2}$ , along with $\mathrm{Cor}\left(X^{*},\;\eta \right)$ . These estimates rely on the point estimates of τ, λ, and ψ, as shown in Equations 2 and 3. In practice, when researchers use Equations 2 and 3, the estimates of the factor model are treated as fixed, but these estimates have standard errors and suffer from sampling variability. Consequently, the estimation of CA and CC ignores the uncertainty of the factor model parameters from the previous step. Although this might not be an issue in simple problems with a large sample size, ignoring the uncertainty of the factor model parameters might be consequential during the estimation of the uncertainty of the CA and CC indices (Levy & Mislevy, 2017), leading to artificially narrow confidence intervals for CA and CC. A possible solution is to turn to bootstrapping and Bayesian methods, which provide natural approaches to acknowledge the uncertainty in the factor model parameters on the estimation of CA and CC summary intervals. Below, bootstrapping and Bayesian methods are discussed.

## Percentile Bootstrap for Confidence Intervals

A motivation for bootstrapping methods is that it is better to estimate confidence intervals using the empirical distribution of a test statistic rather than its theoretical distribution (Efron & Tibshirani, 1993), especially when the theoretical distribution of a test statistic deviates from normality. To approximate the empirical distribution of a test statistic using the bootstrap, one resamples the dataset with replacement (i.e., the same case can appear multiple times on each resample) many times (e.g., 500 or more times), the test statistic is estimated in each of the resampled datasets, and the empirical distribution of the test statistic is derived by plotting or taking summary statistics of all the estimates across resampled datasets. Then, confidence intervals can be derived using percentiles from the empirical distribution. For example, a 95% percentile bootstrap confidence interval would be defined by the 2.5th and the 97.5th percentiles of the empirical distribution. Recall that confidence intervals describe the long-run frequency of covering the true value of the parameter if confidence intervals were to be repeatedly and identically constructed.

## Bayesian Estimation for Credible Intervals

To introduce Bayesian methods, it is important to contrast their focus with frequentist methodology, which tends to dominate in the social sciences. Frequentist methods assume that the data are a random sample from the population and the parameters we estimate (e.g., factor loadings, item intercepts, etc.) are fixed, so we are interested in the magnitude of the point estimate and its standard error. In Bayesian methods, data are treated as fixed observations and the parameters estimated are treated as random, so the parameters take probability distributions (Levy & Mislevy, 2017). Before the analysis, parameters are assigned prior distributions that encode the knowledge we have about the parameters. The information about the parameters can be obtained from multiple sources, such as estimates from pilot data, results from a meta-analysis, eliciting information from subject matter experts, or a combination of all (van de Schoot et al., 2021). Users specify the distribution per parameter (e.g., normal, inverse gamma, etc.) and numerical values that govern the shape of the distribution, and consequently the amount of information they have. For the estimation of the CA and CC indices, one needs to specify priors for the factor loadings, item intercepts, and residual variances. Suppose that a researcher assigns a prior normal distribution with a mean of .5 and a standard deviation of 1 to a factor loading. In this case, researchers are effectively encoding their belief that the likely values for the factor loading to be between −1.5 and 2.5 (which are, roughly, the 2.5th and 97.5th percentiles of that prior normal distribution). Perhaps there are applications in which a meta-analysis suggests that the factor loading is unlikely to be negative, so one could specify a prior distribution in which the likely values are all positive, such as a normal distribution with a mean of .5 and a standard deviation of .2. In the case of pilot data, estimates and standard errors of the parameters of interest can be translated into priors, where priors are centered in the estimates and the variability of the prior is a function of the standard error of the estimate. In situations in which information is obtained by eliciting expert information, there are many tools and protocols to facilitate the process, and these are discussed elsewhere (see Veen et al., 2017 and references therein). Overall, the inclusion of informative priors based on pilot data, meta-analyses, or expert elicitation is a strength of the Bayesian methods. In situations in which there is a lot of uncertainty about the parameters, then diffused prior distributions, prior distributions in which parameters can have a large range of values, or weakly informative distributions, informative priors as discussed above but with added uncertainty, are assigned.

Once prior distributions are assigned, the knowledge of the parameters is updated with observed data using Bayes’ theorem. Bayes’ theorem indicates that p(θ|data) , the posterior distribution of the parameter *θ* after observing the data, is the product of the likelihood of observing the data given the parameter, p(data|θ) , and the prior distribution of the parameter, p(θ) , divided by the marginal probability of observing the data, p(data) ,

(5) $$p\left(\theta |\mathrm{data}\right)=\frac{p\left(\mathrm{data}|\theta \right)p\left(\theta \right)}{p\left(\mathrm{data}\right)},$$

which reduces to p(θ|data)∝p(data|θ)p(θ) because p(data) remains the same in all the analyses. Finally, Markov Chain Monte Carlo (MCMC) estimation is used to combine the likelihood and the prior to approximate the posterior distribution. In general, MCMC estimation can be thought of as a set of sampling algorithms used to sample from a posterior distribution, and in this case, a Gibbs sampler was used (Casella & George, 1992).

Inferences about the parameters are then based on draws from the posterior distributions. The posterior distribution can be described using central tendency and summary intervals. For central tendency, one could use the mean or the median of the distribution. Summary intervals could either be equal-tail or highest posterior density (HPD) intervals. Equal-tail intervals capture the specified density and are balanced so that the excluded density is the same above the upper limit and below the lower limit. For example, a 95% credible interval is defined by the 2.5th and the 97.5th percentiles of the posterior distribution. However, HPD intervals are constructed iteratively so that they capture the specified density, but they are not balanced (Gelman et al., 2004)—their property is that no value outside of the interval for the posterior distribution has a higher probability than the value inside of the HPD interval. Although HPD intervals are more common to summarize posterior distributions, similar procedures could be used to summarize empirical distributions from the bootstrap. Finally, the interpretation of credible intervals differs from the confidence intervals described above—credible intervals describe the probability that the true parameter is within the range of values.

## How Bootstrapping and Bayesian Methods Handle Uncertainty of Derived Quantities?

An advantage of estimating summary intervals with bootstrapping and Bayesian methods is the calculation of uncertainty of derived quantities that are functions of other parameters. Bootstrapping methods incorporate the uncertainty of the factor model parameters in the summary intervals by (a) estimating the factor model in each of the bootstrap datasets, (b) saving the parameters of the linear factor model per dataset, (c) calculating $\mu_{X^{*}},\;\sigma_{X^{*}}^{2},$ and $\mathrm{Cor}\left(X^{*},\;\eta \right)$ from Equations 2 and 3, (d) defining the bivariate normal distribution, (e) estimating CA and CC per dataset, and (f) collecting the estimates to derive the empirical distributions of CA and CC. Those empirical distributions of CA, sensitivity, specificity, and classification consistency could then be summarized with central tendency and summary intervals. In each bootstrapped dataset, the estimates of the linear factor model will vary, reflecting their sampling variability. However, Bayesian methods accomplish the incorporation of uncertainty by using the posterior distribution of the estimated factor model parameters, not just the point estimates, in the estimation of posterior distributions for CA and CC indices. In essence, one would estimate the linear factor model using MCMC estimation and posterior distributions per parameter would be obtained (Edwards, 2010). Then, at each MCMC iteration, we would use each set of draws from each of the posterior distributions to calculate $\mu_{X^{*}},\;\sigma_{X^{*}}^{2},$ and $\mathrm{Cor}\left(X^{*},\;\eta \right)$ from Equations 2 and 3, define the bivariate normal distributions, and calculate the CA and CC indices. Ultimately, there would be posterior distributions for the classification rate, sensitivity, specificity, and classification consistency, which we could summarize using central tendency and summary intervals. As such, the uncertainty in the factor model parameters is propagated to the empirical distribution or the posterior distribution of CA and CC, and thus acknowledged in their summary intervals.

## Present Study

So far, two approaches to estimate summary intervals for model-based CA and CC indices that accommodate the propagation of uncertainty from the factor model parameters to the CA and CC estimates have been introduced. Below, the estimation and interpretation of bootstrap and Bayesian summary intervals using an applied example are demonstrated. Also, simulation results on the bias and coverage of bootstrap and Bayesian summary intervals for the CA and CC indices are discussed to provide general recommendations. Similar to prior research (Pfadt et al., 2021), the summary intervals are expected to be centered around the true value (i.e., median estimate would have low bias) and have close to a 95% coverage, although coverage might be slightly underestimated in conditions with small sample sizes. Also, it is expected that summary interval width would decrease as the sample size increases.

## Illustration

### Data

The dataset for this illustration is from a study by Eisenberg et al. (2018) on the measurement of the self-regulation construct. The dataset had *N* = 522 Mturk participants who responded to a large battery of self-regulation measures. All participants passed a basic validity check to filter out those who were giving bogus responses. Out of the respondents in the sample, 50.2% were female, 78.8% were White, and 83.9% had at least a college education. Among the battery of measures, participants responded to the Mindful Attention Awareness Scale (MAAS; Brown & Ryan, 2003), which is a 15-item measure that assesses one’s tendency to be fully aware of their experience in the moment without distraction. Items are rated on a 6-point Likert-type scale ranging from 1 (*almost always*) to 6 (*almost never*), so the range from possible summed scores is 15 to 90. Items include, “I snack without being aware that I’m eating” and “I do jobs or tasks automatically, without being aware of what I’m doing.” Items were recoded so that higher scores represented more mindfulness, and the summed score reliability was α = .92. Suppose that we were interested in identifying respondents who are low on mindfulness, which is defined as being at −1 *SD* or below the mindfulness construct, to enroll them in an intervention. Ideally, we would choose individuals based on the latent variable score with a cutpoint of −1 (e.g., η≤−1 *SD*) that yields true classifications, however, the summed score, which is prone to measurement error, is commonly used in practice to select individuals (e.g., $X^{*}$ ≤−1 *SD*, corresponding to $X^{*}$ ≤ 53). The goal of this illustration is to estimate CA and CC of the MAAS for those respective cutpoints, along with both bootstrapped and Bayesian summary intervals for the indices.

### Procedure

First, a unidimensional factor model was fit to the data using lavaan (Rosseel, 2012) in R, and model fit was reported, and CA and CC indices were estimated as outlined above. Then, to estimate the bootstrapped confidence intervals, 500 datasets with replacement were sampled, a unidimensional factor model was fit to each bootstrapped dataset using lavaan, and factor model parameters for each bootstrapped dataset were saved. Then, for each set of factor model parameters, Equations 2 and 3 were used to determine $\mu_{X^{*}}$ , $\sigma_{X^{*}}^{2}$ , and $\mathrm{Cor}\left(X^{*},\;\eta \right)$ , respectively. Next, two bivariate normal distributions were derived. First, the bivariate normal distribution of $X^{*}$ and η was used to estimate the CA indices, to which the cutpoints on $X^{*}$ and η were imposed to create four quadrants. Then, the proportion of each quadrant was estimated via integration using the pmvnorm function in the mvtnorm R-package. Second, the bivariate normal distribution of $X_{1}^{*}$ and $X_{2}^{*}$ , in which $\mathrm{Cor}\left(X_{1}^{*},\;X_{2}^{*}\right)$ was $\mathrm{Cor}{\left(X^{*},\;\eta \right)}^{2}$ , to which the cutpoints on $X^{*}$ were imposed, was used to estimate the CC index. Similar steps were taken to determine the proportion of each quadrant. Finally, we use the proportion of each quadrant to estimate the classification rate, sensitivity, specificity, and classification consistency as indicated above. Overall, there were 500 values of the CA and CC indices, which were summarized using the median and a 95% equal-tail interval and a 95% HPD interval using the coda package.

To estimate the Bayesian summary intervals, we fit a unidimensional factor model to the data using JAGS (Plummer, 2003) via R using the R2jags package (Su & Yajima, 2020). The full posterior distribution for the unidimensional factor model is,

$$\begin{array}{cc} & p\left(\eta ,\tau ,\lambda ,\;\psi |x\right)=\;\overset{J}{\underset{j=1}{\Pi }}\overset{N}{\underset{n=1}{\Pi }}p\left(x_{\mathrm{ij}}|\eta_{i},\;\tau_{j},\;\lambda_{j},\;\psi_{\mathrm{jj}}\right)p\left(\eta |\kappa ,\Phi \right)p\left(\lambda_{j}\right)p\left(\tau_{j}\right)p\left(\psi_{\mathrm{jj}}\right),\;\mathrm{where}\;\\ & x_{\mathrm{ij}}\;|\;\eta_{i},\;\tau_{j},\;\lambda_{j},\;\psi_{\mathrm{jj}}\;\sim \;N\left(\tau_{j}+\lambda_{j}\eta_{i},\;\psi_{j}\right),\\ \eta_{i},|\kappa ,\Phi \sim N(\kappa ,\Phi ),\mathrm{where}\;\kappa =0,\Phi =1,\\ & \tau_{j}\sim N\left(3,\;10\right),\;\\ \lambda_{j}\sim \;N\left(1,\;10\right),\;\mathrm{and}\\ \psi_{j}=\mathrm{IG}\left(5,\;10\right). \end{array}$$

In this case, $\tau_{j}$ , $\lambda_{j}$ , and $\psi_{j}$ had diffused priors relative to the scale of the items— $\tau_{j}$ had priors that were normally distributed with mean of 3 and a standard deviation of 10, $\lambda_{j}$ had priors that were normally distributed with a mean of one and a standard deviation of 10, and $\psi_{j}$ had an inverse gamma distribution, which is a popular distribution for (residual) variances, with a shape parameter of 5 and a scale parameter of 10. In this case, the parameters of the inverse gamma roughly translate to residuals variances around 2 with not a lot of certainty, as if the estimate came from a sample of 10 individuals (Levy & Mislevy, 2017). Note that there are no prior distributions for κ or Φ , which are set 0 and 1, respectively, for identification.

For the MCMC implementation, there were three chains which each drew 5,000 iterations. The first 1,000 were burn-in iterations and were discarded, and 4,000 post-burn-in iterations per chain were examined. We specified a thinning of 4, so every fourth iteration was saved for further analyses, yielding 3,000 post-thinning iterations (i.e., 1,000 per chain) that defined the posterior distributions of each parameter. Trace plots and the potential scale reduction factor (PSRF; Brooks & Gelman, 1998) were used as evidence of convergence of the chains, where the PSRF criteria was < 1.1. Finally, each set of draws per iteration (e.g., first draw of $\tau_{1}$ , $\tau_{2}$ , . . ., $\lambda_{1}$ , $\lambda_{2}$ , . . ., $\psi_{1}$ , $\psi_{2}$ ) were taken to estimate $\mu_{X^{*}}$ , $\sigma_{X^{*}}^{2}$ , along with $\mathrm{Cor}\left(X^{*},\;\eta \right)$ , and conduct the procedure described above to estimate the CA and CC indices, which were summarized using the median, a 95% equal-tail interval, and a 95% HPD interval using the coda package (Plummer et al., 2006).

### Results

**Figure 2.**
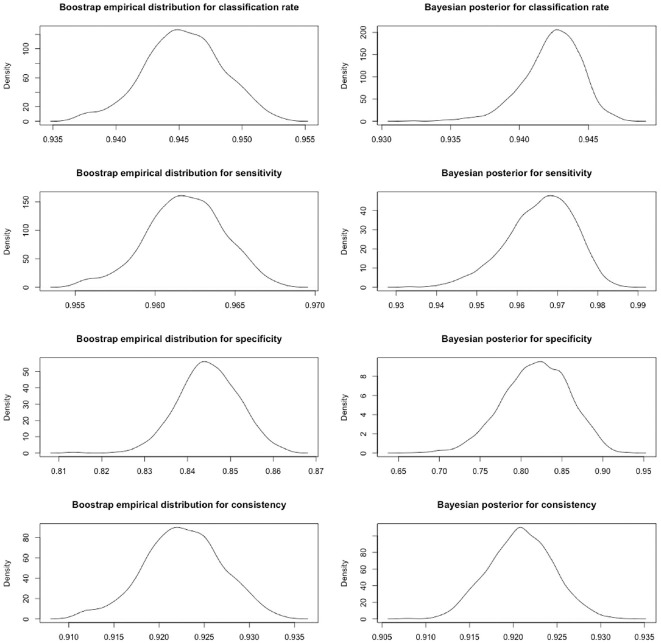
Density Plots for the Classification Accuracy and Consistency Indices Estimated with the Bootstrap or Bayesian Estimation

Model fit statistics suggest that the unidimensional factor model fits the 15 MAAS items generally well, χ^2^(90) = 494.214, *p*< .001, comparative fit index (CFI) = .924, root mean square error of approximation (RMSEA) = .079, standardized root mean square residual (SRMR) = .042. For MCMC estimation, the PSRF for each factor model parameter suggests that the chains converged to the same distribution. Parameter estimates for the factor loadings, intercepts, and residual variances are shown in the supplement. Results suggest that the Bayesian equal-tail intervals, the Bayesian HPD intervals, and their bootstrap counterparts were similar to each other (see Table 1). Figure 2 shows the density plots of the empirical distribution using the bootstrap and the Bayesian posterior distributions for the CA and CC estimates, which are unimodal. Here, the equal-tail summary intervals of the CC index are interpreted, and the rest of the estimates have a similar interpretation. For the bootstrap, median of the empirical distribution of classification consistency was .923, with a 95% equal-tail confidence interval of [.914, .931], which provides an idea of the sampling variability of the estimate. For the Bayesian credible intervals, the median of the posterior distribution of classification consistency was .921, with a 95% equal-tail credible interval of [.914, .928], which means that there is a 95% probability that classification consistency is between .914 and .928. By reporting summary intervals of the CA and CC indices, the uncertainty of those estimates is acknowledged.

**Table 1.** Classification Accuracy and Consistency Indices to Select Respondents Who Are 1 SD or Below on Mindfulness.

	Median	2.5th EQ	97.5th EQ	LL HPD	UP HPD
Bootstrap summary intervals					
Classification rate	0.945	0.939	0.951	0.939	0.951
Sensitivity	0.962	0.957	0.966	0.957	0.966
Specificity	0.845	0.831	0.858	0.831	0.858
Consistency	0.923	0.914	0.931	0.914	0.931
Bayesian summary intervals					
Classification rate	0.943	0.938	0.946	0.938	0.946
Sensitivity	0.967	0.947	0.979	0.949	0.980
Specificity	0.820	0.736	0.891	0.743	0.896
Consistency	0.921	0.914	0.928	0.914	0.928

*Note.* EQ = equal-tail; LL = lower limit; HPD = highest posterior density; UP = upper limit.

## Simulation Study

### Simulation Factors and Procedure

For the simulation, datasets were generated in R based on a unidimensional linear factor model and three factors were varied: sample size (*N* = 200 and 500), the number of items (*i* = 5, 10, 20), and the average factor loading between the indicators and the latent variable (*f* = .5, .6, and .7). The factor mean and variance were set to zero and one, respectively, and the true factor loadings were drawn from a uniform distribution centered at *f* and limits at ± .2 (see Table A0 in supplementary materials for these values). Furthermore, the item intercepts for all the items were specified to be zero, and the residual variances were specified so that the total variance of the indicator was 1 (i.e., residual variances were 1-*f*^2^). Although the number of items and the magnitude of the factor loadings are expected to affect the CA and CC estimates, the sample size is expected to affect the sampling variability of the factor model parameters.

The general steps for data analysis, computation of classification rate, sensitivity, specificity, and classification consistency indices, and estimation of summary intervals were largely similar to those described in the illustration. Three approaches were examined: bootstrap confidence intervals, Bayesian credible intervals with diffused priors, and (for illustrative purposes) Bayesian credible intervals with empirical, weakly informative priors. Diffused priors were similar to those used in the illustration. For the analyses with empirical, weakly informative priors, the prior for the loadings and intercepts was a normal distribution centered at the empirical value obtained by fitting a unidimensional model to the data using maximum likelihood, and the standard deviation was 1. Note that there is added uncertainty in the prior distributions about the parameters, which is larger than the uncertainty reflected in the standard errors of the maximum likelihood solution. Similarly, for the residual variances, a prior inverse gamma distribution that reflects a variance similar to the empirical values obtained and with a pseudo sample size of 20 was used.^2^ For each method, a 95% equal-tail summary interval and a 95% HPD summary interval were estimated using three sets of cutpoints: both the expected summed score and η with a cutpoint at the mean, 0.75 *SD* above the mean, and 1.5 *SD* about the mean.^3^ The cutpoint for the summed score was estimated using the implied moments of data-generating item parameters, so the cutpoints were fixed across replications (e.g., the cutpoints were not estimated again). Overall, there were 18 conditions (2 Sample Sizes × 3 Average Loadings × 3 Numbers of Items), with 1,000 replications per condition, per index (4; sensitivity, specificity, classification rate, and classification consistency), cutpoint (3; mean, .75 *SD*, 1.5 *SD*), type of interval (2; equal-tail or HPD), and method to estimate summary intervals (3; bootstrap, Bayesian diffused, Bayesian empirical, weakly informative), so there a total of 1,296,000 summary intervals examined.

### Simulation Outcomes

The true values of the CA and CC indices were determined with Equations 2 and 3 and are shown in the supplementary materials (see Table A1). Based on those true values, the simulation outcomes are the relative bias of the CA and CC point estimates and summary interval coverage. Relative bias was estimated by subtracting the median estimate of the CA and CC indices per replication from the true value and dividing by the true value. A relative bias between .025 and .075 would be deemed acceptable. Summary interval coverage was estimated by the number of times that the true value was within the summary interval limits. A summary interval coverage between .925 and .975 would be deemed acceptable. The average width and the balance of each summary interval (i.e., proportion of times that the estimate was to the left or right of the summary interval limits) were also reported. Note that the true CA and CC indices vary by the cutpoint, average factor loading, and the number of items, so we describe the performance of the summary intervals within each condition, rather than comparing across conditions.

### Results

#### Bootstrap Summary Intervals

Largely, bootstrap summary intervals exhibited the best performance out of the summary intervals studied. For conditions with a cutpoint at the mean of the summed score and latent variable, the median estimate of the bootstrap empirical distribution across CA and CC indices had a relative bias within ± .05 (see Table A2 in the supplement). Regarding summary interval coverage, both 95% equal-tail and 95% HPD bootstrap summary intervals had coverage between .925 and .975 in all but five conditions (in which coverage was .890; see Table 2). The bootstrap summary intervals were generally balanced for sensitivity, specificity, and classification consistency, but there was right imbalance for the classification rate—the true value of classification rate was consistently above the upper limit of the interval. Also, bootstrap summary interval width decreased as sample size increased (see Table A3 in the supplement). Similar findings were also observed for conditions in which the cutpoint was at .75 *SD* (see Tables A4 and A5 in supplement). For conditions in which the cutpoint was 1.5 *SD*, most of the summary intervals for the classification rate had coverage above .975, suggesting that the summary intervals were unnecessarily wide (see Tables A6 and A7 in supplement). For sensitivity, specificity, and classification consistency, there were 14 summary intervals with coverage between .90 and .925, out of which 12 of those conditions were those with HPD summary intervals.

**Table 2.** Bootstrap Confidence Interval Coverage and Balance [Lower, Upper] for a Cutpoint at the Mean.

i	*n*	95% Equal-tail bootstrap confidence interval			95% HPD bootstrap confidence interval		
		*f* = .5	*f* = .6	*f* = .7	*f* = .5	*f* = .6	*f* = .7
*Classification rate*							
5	200	0.949[0.010,0.041]	0.943[0.004,0.053]	0.941[0.001,0.058]	0.955[0.012,0.033]	0.949[0.005,0.046]	0.948[0.001,0.051]
	500	0.948[0.002,0.050]	**0.922[0.000,0.078]**	**0.897[0.000,0.103]**	0.951[0.003,0.046]	0.935[0.000,0.065]	0.937[0.000,0.063]
10	200	0.942[0.001,0.057]	0.930[0.000,0.070]	**0.917[0.000,0.083]**	0.953[0.006,0.041]	0.956[0.002,0.042]	0.944[0.000,0.056]
	500	0.946[0.019,0.035]	0.955[0.009,0.036]	0.946[0.004,0.050]	0.945[0.023,0.032]	0.957[0.009,0.034]	0.948[0.006,0.046]
20	200	0.954[0.005,0.041]	0.945[0.005,0.050]	0.943[0.001,0.056]	0.957[0.004,0.039]	0.949[0.008,0.043]	0.963[0.001,0.036]
	500	0.957[0.003,0.040]	0.965[0.001,0.034]	0.938[0.000,0.062]	0.959[0.004,0.037]	0.971[0.002,0.027]	0.957[0.000,0.043]
*Sensitivity*							
5	200	0.957[0.022,0.021]	0.931[0.030,0.039]	0.944[0.025,0.031]	0.941[0.035,0.024]	**0.922[0.043,0.035]**	0.936[0.035,0.029]
	500	0.947[0.025,0.028]	0.949[0.021,0.030]	0.950[0.024,0.026]	0.942[0.033,0.025]	0.940[0.036,0.024]	0.944[0.033,0.023]
10	200	0.934[0.026,0.040]	0.947[0.027,0.026]	0.949[0.024,0.027]	**0.920[0.043,0.037]**	0.936[0.041,0.023]	0.933[0.045,0.022]
	500	0.950[0.026,0.024]	0.951[0.021,0.028]	0.942[0.020,0.038]	0.946[0.031,0.023]	0.953[0.022,0.025]	0.936[0.032,0.032]
20	200	0.943[0.018,0.039]	0.940[0.034,0.026]	0.950[0.021,0.029]	0.944[0.022,0.034]	0.936[0.042,0.022]	0.945[0.028,0.027]
	500	0.961[0.019,0.020]	0.950[0.023,0.027]	0.951[0.026,0.023]	0.955[0.025,0.020]	0.945[0.032,0.023]	0.939[0.039,0.022]
*Specificity*							
5	200	0.948[0.020,0.032]	0.938[0.030,0.032]	0.945[0.033,0.022]	0.943[0.030,0.027]	0.931[0.042,0.027]	0.936[0.045,0.019]
	500	0.942[0.023,0.035]	0.947[0.018,0.035]	0.950[0.021,0.029]	0.938[0.034,0.028]	0.946[0.024,0.030]	0.934[0.039,0.027]
10	200	0.949[0.027,0.024]	0.946[0.026,0.028]	0.944[0.023,0.033]	0.942[0.036,0.022]	0.934[0.041,0.025]	0.937[0.041,0.022]
	500	0.951[0.020,0.029]	0.957[0.016,0.027]	0.959[0.023,0.018]	0.940[0.031,0.029]	0.951[0.027,0.022]	0.953[0.031,0.016]
20	200	0.950[0.025,0.025]	0.937[0.024,0.039]	0.951[0.025,0.024]	0.942[0.035,0.023]	0.934[0.028,0.038]	0.944[0.036,0.020]
	500	0.957[0.022,0.021]	0.942[0.031,0.027]	0.952[0.023,0.025]	0.945[0.036,0.019]	0.938[0.040,0.022]	0.950[0.029,0.021]
*Classification consistency*							
5	200	0.945[0.032,0.023]	0.944[0.031,0.025]	0.949[0.021,0.030]	0.944[0.041,0.015]	0.938[0.040,0.022]	0.950[0.027,0.023]
	500	0.954[0.025,0.021]	0.947[0.016,0.037]	0.949[0.024,0.027]	0.949[0.034,0.017]	0.945[0.020,0.035]	0.945[0.030,0.025]
10	200	0.948[0.025,0.027]	0.951[0.029,0.020]	0.951[0.009,0.040]	0.945[0.032,0.023]	0.942[0.040,0.018]	0.941[0.021,0.038]
	500	0.940[0.037,0.023]	0.960[0.019,0.021]	0.947[0.023,0.030]	0.941[0.040,0.019]	0.955[0.025,0.020]	0.940[0.032,0.028]
20	200	0.958[0.020,0.022]	0.954[0.021,0.025]	0.966[0.016,0.018]	0.950[0.029,0.021]	0.949[0.027,0.024]	0.969[0.014,0.017]
	500	0.954[0.017,0.029]	0.967[0.018,0.015]	0.969[0.008,0.023]	0.949[0.024,0.027]	0.963[0.022,0.015]	0.963[0.011,0.026]

*Note. i* is for number of items, *n* is for sample size, and *f* is for average factor loading, and in bold are intervals outside the robustness criterion. HPD = highest posterior density.

#### Bayesian Summary Intervals With Diffused Priors

Across conditions, the Bayesian summary intervals with diffused priors exhibited poor performance. The summary intervals coverage for classification rate and classification consistency were well below .925, with as low as .008, across all cutpoints (see Table 3 for the cutpoint at mean, and Tables B2 and B4 in the supplement for cutpoints at .75 *SD* and 1.5 *SD*). There was noticeable right imbalance for the summary intervals, which maps on to the negative relative bias in the median estimates (see Tables B1, B3, and B5 in supplement). There were some conditions in which the summary intervals for sensitivity and specificity were within .925 and .975 (still exhibiting right imbalance), but those are not further discussed given the poor summary interval performance for classification rate and consistency.

**Table 3.** Bayesian Credible Interval Coverage (Diffused Priors) and Balance [Lower, Upper] for a Cutpoint at the Mean.

i	* n*	95% Equal-tail Bayesian credible interval			95% HPD Bayesian credible interval		
		*f* = .5	*f* = .6	*f* = .7	*f* = .5	*f* = .6	*f* = .7
*Classification rate*							
5	200	**0.335[0.000,0.665]**	**0.130[0.000,0.870]**	**0.008[0.000,0.992]**	**0.355[0.000,0.645]**	**0.145[0.000,0.855]**	**0.011[0.000,0.989]**
	500	**0.537[0.000,0.463]**	**0.346[0.000,0.654]**	**0.109[0.000,0.891]**	**0.549[0.000,0.451]**	**0.370[0.000,0.630]**	**0.167[0.000,0.833]**
10	200	**0.800[0.000,0.200]**	**0.710[0.000,0.290]**	**0.465[0.000,0.535]**	**0.805[0.000,0.195]**	**0.755[0.000,0.245]**	**0.549[0.000,0.451]**
	500	**0.620[0.000,0.380]**	**0.389[0.000,0.611]**	**0.158[0.000,0.842]**	**0.633[0.000,0.367]**	**0.409[0.000,0.591]**	**0.165[0.000,0.835]**
20	200	**0.742[0.000,0.258]**	**0.623[0.000,0.377]**	**0.364[0.000,0.636]**	**0.745[0.000,0.255]**	**0.626[0.000,0.374]**	**0.473[0.000,0.527]**
	500	**0.879[0.001,0.120]**	**0.844[0.000,0.156]**	**0.687[0.000,0.313]**	**0.868[0.001,0.131]**	**0.845[0.000,0.155]**	**0.701[0.000,0.299]**
*Sensitivity*							
5	200	**0.864[0.001,0.135]**	**0.836[0.002,0.162]**	**0.850[0.001,0.149]**	**0.874[0.001,0.125]**	**0.852[0.004,0.144]**	**0.869[0.001,0.130]**
	500	**0.922[0.002,0.076]**	0.928[0.004,0.068]	0.925[0.003,0.072]	0.925[0.005,0.070]	0.930[0.006,0.064]	0.937[0.004,0.059]
10	200	0.945[0.010,0.045]	0.959[0.010,0.031]	0.955[0.007,0.038]	0.944[0.015,0.041]	0.960[0.016,0.024]	0.957[0.015,0.028]
	500	**0.907[0.005,0.088]**	**0.912[0.003,0.085]**	**0.907[0.000,0.093]**	**0.908[0.006,0.086]**	**0.919[0.003,0.078]**	**0.913[0.003,0.084]**
20	200	0.940[0.006,0.054]	0.934[0.016,0.050]	0.945[0.006,0.049]	0.940[0.007,0.053]	0.938[0.017,0.045]	0.949[0.006,0.045]
	500	0.967[0.007,0.026]	0.950[0.015,0.035]	0.951[0.012,0.037]	0.966[0.010,0.024]	0.950[0.019,0.031]	0.948[0.023,0.029]
*Specificity*							
5	200	**0.855[0.001,0.144]**	**0.850[0.001,0.149]**	**0.817[0.004,0.179]**	**0.862[0.001,0.137]**	**0.855[0.003,0.142]**	**0.835[0.005,0.160]**
	500	**0.923[0.001,0.076]**	0.930[0.001,0.069]	0.925[0.004,0.071]	0.930[0.003,0.067]	0.940[0.004,0.056]	0.933[0.005,0.062]
10	200	0.961[0.007,0.032]	0.950[0.007,0.043]	0.952[0.006,0.042]	0.956[0.014,0.030]	0.952[0.019,0.029]	0.962[0.012,0.026]
	500	**0.907[0.000,0.093]**	**0.899[0.003,0.098]**	**0.908[0.003,0.089]**	**0.909[0.001,0.090]**	**0.906[0.005,0.089]**	**0.913[0.006,0.081]**
20	200	0.944[0.009,0.047]	0.924[0.011,0.065]	0.941[0.011,0.048]	0.947[0.010,0.043]	0.926[0.014,0.060]	0.943[0.015,0.042]
	500	0.968[0.011,0.021]	0.953[0.013,0.034]	0.951[0.017,0.032]	0.963[0.015,0.022]	0.947[0.023,0.030]	0.952[0.021,0.027]
*Classification consistency*							
5	200	**0.434[0.000,0.566]**	**0.224[0.000,0.776]**	**0.023[0.000,0.977]**	**0.465[0.000,0.535]**	**0.230[0.000,0.770]**	**0.025[0.000,0.975]**
	500	**0.669[0.000,0.331]**	**0.501[0.000,0.499]**	**0.247[0.000,0.753]**	**0.665[0.000,0.335]**	**0.490[0.000,0.510]**	**0.244[0.000,0.756]**
10	200	**0.898[0.000,0.102]**	**0.836[0.002,0.162]**	**0.648[0.000,0.352]**	**0.898[0.000,0.102]**	**0.833[0.002,0.165]**	**0.629[0.000,0.371]**
	500	**0.712[0.000,0.288]**	**0.483[0.000,0.517]**	**0.230[0.000,0.770]**	**0.722[0.000,0.278]**	**0.490[0.000,0.510]**	**0.220[0.000,0.780]**
20	200	**0.806[0.001,0.193]**	**0.732[0.000,0.268]**	**0.582[0.000,0.418]**	**0.801[0.001,0.198]**	**0.715[0.000,0.285]**	**0.564[0.000,0.436]**
	500	**0.915[0.001,0.084]**	**0.911[0.001,0.088]**	**0.788[0.000,0.212]**	**0.898[0.002,0.100]**	**0.897[0.001,0.102]**	**0.751[0.000,0.249]**

*Note. i* is for number of items, *n* is for sample size, and *f* is for average factor loading, and in bold are intervals outside the robustness criterion. HPD = highest posterior density.

#### Bayesian Summary Intervals With Empirical, Weakly Informative Priors

There is better coverage and narrower credible interval width by shifting from diffused priors to empirical, weakly informative priors. Across conditions, most of the median estimates for CA and CC indices had a relative bias within ± .05 (see Table C1, C3, and C5 in the supplement). This finding is not surprising given that the prior distributions of factor model parameters were centered on their empirical values estimated via maximum likelihood. Regarding summary interval coverage, all credible intervals for sensitivity and specificity with a cutpoint at the mean had coverage between .925 and .975. There were 10 conditions with a cutpoint at the mean in which there were credible intervals for classification rate were above .975 (i.e., unnecessarily large). Also, credible intervals for classification consistency in conditions with a sample size of 200 and 10 items had credible intervals below .925 (with the lowest at .874). Similar findings regarding classification rate having unnecessarily wide credible intervals were observed in conditions with cutpoints of 0.75 *SD* and 1.5 SD (see Tables C2 and C4). However, sensitivity had credible intervals below .925, while specificity and classification consistency had most of their credible intervals within .925 and .975.

**Table 4.** Bayesian Credible Interval Coverage (Informative Priors) and Balance [Lower, Upper] for a Cutpoint at the Mean.

i	* n*	95% Equal-tail Bayesian credible interval			95% HPD Bayesian credible interval		
		*f* = .5	*f* = .6	*f* = .7	*f* = .5	*f* = .6	*f* = .7
*Classification rate*							
5	200	0.952[0.014,0.034]	0.953[0.005,0.042]	0.962[0.000,0.038]	0.956[0.016,0.028]	0.958[0.007,0.035]	0.966[0.000,0.034]
	500	0.971[0.007,0.022]	0.966[0.000,0.034]	0.961[0.000,0.039]	0.970[0.008,0.022]	0.970[0.000,0.030]	**0.982[0.000,0.018]**
10	200	0.976[0.017,0.007]	**0.987[0.002,0.011]**	**0.980[0.000,0.020]**	0.972[0.021,0.007]	**0.992[0.006,0.002]**	**0.988[0.000,0.012]**
	500	0.947[0.021,0.032]	0.961[0.008,0.031]	0.954[0.004,0.042]	0.949[0.021,0.030]	0.963[0.011,0.026]	0.955[0.005,0.040]
20	200	0.969[0.010,0.021]	0.962[0.010,0.028]	0.963[0.001,0.036]	0.967[0.012,0.021]	0.966[0.012,0.022]	**0.982[0.001,0.017]**
	500	0.972[0.019,0.009]	**0.984[0.003,0.013]**	**0.981[0.002,0.017]**	0.970[0.018,0.012]	**0.982[0.006,0.012]**	**0.983[0.002,0.015]**
*Sensitivity*							
5	200	0.959[0.021,0.020]	0.935[0.032,0.033]	0.951[0.024,0.025]	0.956[0.027,0.017]	0.931[0.040,0.029]	0.940[0.036,0.024]
	500	0.950[0.031,0.019]	0.953[0.024,0.023]	0.959[0.022,0.019]	0.947[0.037,0.016]	0.947[0.036,0.017]	0.946[0.037,0.017]
10	200	0.941[0.037,0.022]	0.959[0.027,0.014]	0.955[0.031,0.014]	0.933[0.048,0.019]	0.941[0.048,0.011]	0.931[0.056,0.013]
	500	0.959[0.025,0.016]	0.960[0.018,0.022]	0.949[0.021,0.030]	0.952[0.031,0.017]	0.953[0.025,0.022]	0.938[0.033,0.029]
20	200	0.950[0.020,0.030]	0.946[0.034,0.020]	0.954[0.021,0.025]	0.951[0.022,0.027]	0.939[0.041,0.020]	0.948[0.029,0.023]
	500	0.967[0.021,0.012]	0.954[0.022,0.024]	0.954[0.026,0.020]	0.962[0.028,0.010]	0.948[0.031,0.021]	0.942[0.042,0.016]
*Specificity*							
5	200	0.955[0.018,0.027]	0.945[0.031,0.024]	0.956[0.027,0.017]	0.947[0.029,0.024]	0.938[0.042,0.020]	0.943[0.041,0.016]
	500	0.950[0.029,0.021]	0.955[0.021,0.024]	0.960[0.019,0.021]	0.950[0.033,0.017]	0.952[0.027,0.021]	0.951[0.034,0.015]
10	200	0.953[0.033,0.014]	0.953[0.033,0.014]	0.963[0.025,0.012]	0.940[0.047,0.013]	0.939[0.049,0.012]	0.944[0.046,0.010]
	500	0.954[0.019,0.027]	0.961[0.017,0.022]	0.959[0.023,0.018]	0.957[0.021,0.022]	0.958[0.022,0.020]	0.957[0.030,0.013]
20	200	0.958[0.024,0.018]	0.943[0.021,0.036]	0.954[0.025,0.021]	0.949[0.034,0.017]	0.942[0.027,0.031]	0.950[0.032,0.018]
	500	0.955[0.029,0.016]	0.946[0.036,0.018]	0.957[0.025,0.018]	0.951[0.037,0.012]	0.940[0.046,0.014]	0.953[0.033,0.014]
*Classification consistency*							
5	200	0.951[0.033,0.016]	0.949[0.035,0.016]	0.949[0.031,0.020]	0.944[0.041,0.015]	0.945[0.039,0.016]	0.944[0.038,0.018]
	500	0.947[0.046,0.007]	0.963[0.028,0.009]	0.950[0.043,0.007]	0.941[0.052,0.007]	0.952[0.037,0.011]	0.948[0.046,0.006]
10	200	**0.897[0.100,0.003]**	**0.882[0.118,0.000]**	**0.906[0.089,0.005]**	**0.881[0.116,0.003]**	**0.874[0.126,0.000]**	**0.894[0.101,0.005]**
	500	0.952[0.035,0.013]	0.966[0.021,0.013]	0.951[0.024,0.025]	0.949[0.039,0.012]	0.963[0.023,0.014]	0.947[0.026,0.027]
20	200	0.957[0.032,0.011]	0.952[0.036,0.012]	0.967[0.022,0.011]	0.950[0.038,0.012]	0.949[0.040,0.011]	0.957[0.032,0.011]
	500	0.943[0.054,0.003]	0.943[0.049,0.008]	0.956[0.038,0.006]	0.934[0.062,0.004]	0.930[0.062,0.008]	0.946[0.047,0.007]

*Note. i* is for number of items, *n* is for sample size, and *f* is for average factor loading, and in bold are intervals outside the robustness criterion. HPD = highest posterior density.

## Discussion

When researchers use scores to make decisions, indices such as the classification rate, sensitivity, specificity, and classification consistency can help describe the quality of the decisions. However, guidance on how to estimate the uncertainty of these CA and CC indices has been limited. In this article, this limitation was addressed by demonstrating how to estimate summary intervals for CA and CC indices by using the percentile bootstrap and Bayesian estimation. Also, the application of summary intervals for CA and CC indices was illustrated using a mindfulness measure and reported results from a small simulation study on the statistical properties of these summary intervals. Generally, simulation results suggest that the percentile bootstrap confidence intervals are unbiased and have coverage close to .95 across most conditions studied. Moreover, the results suggest that Bayesian credible intervals with empirical, weakly informative prior distributions outperform credible intervals with diffused priors regarding coverage, although there were many conditions in which the credible intervals with empirical, weakly informative prior distributions were unnecessarily wide. We do not encourage the use of data-dependent priors for the estimation of summary intervals of CA and CC indices, but a takeaway of the results is that using informative priors (e.g., from pilot data, a meta-analysis, or eliciting information from experts) could potentially improve the coverage of the CA and CC summary intervals. As such, we recommend using equal-tail bootstrap confidence intervals to estimate the uncertainty of the CA and CC indices. Overall, the example, R code, and simulation results provide guidance on how to estimate summary intervals for CA and CC indices and under which conditions the summary intervals work well.

The overarching goal of this article is to encourage researchers to report summary intervals for the CA and CC indices when they use item responses for classification. Note that the CA and CC indices are specific to the cutpoints on $X^{*}$ and η used. Often, researchers use cutpoints that aim to maximize the sensitivity and specificity of the measure, which implicitly weighs false positives and false negatives equally (Youngstrom, 2014). However, in many applications, making a false negative (e.g., failing to treat someone with depression) is riskier than making a false positive (e.g., treating an individual for depression who is not depressed). As such, these risks can be mitigated by shifting the cutpoint and increase (decrease) sensitivity by sacrificing (to gain) specificity (Youngstrom, 2014). Once the application-specific cutpoint is determined, then the CA and CC indices and their summary intervals can be reported.

There are several limitations and future directions to this study. As in any model-based estimate, the estimation of CA and CC indices presumes that the model fits the data well and is correctly specified, but model misspecification was not studied. An important future direction would be to examine how unmodeled multidimensionality (Reise et al., 2013), local dependence (Edwards et al., 2018), or model error (MacCallum et al., 2001) affect the estimation of the CA and CC indices and their summary intervals. Moreover, it would be interesting to study the estimation and summary intervals of model-based positive and negative predictive values (PPV and NPV), which might be more useful than the CA and CC indices to understand respondent-level classifications. In this case, PPV would describe the probability that an individual has the condition as indicated by η if the individual is above the cutpoint on $X^{*}$ , and NPV would describe the probability that an individual does not have the condition as indicated by η if the individual is below the cutpoint on $X^{*}$ . As opposed to CA and CC indices discussed, PPV and NPV depend on the prevalence of the condition based on η studied (Pepe, 2003). Furthermore, the simulation study only included a limited set of prior distributions for the Bayesian summary intervals, which affects the overall conclusion of the study. Future directions should determine how to best obtain and encode prior information for the factor model, which consequently affects the CA and CC indices, along with studying the sensitivity of the results to specific priors (van Erp et al., 2018). Also, there are model-based procedures to estimate CA and CC indices for items that do not fit the linear factor model (i.e., binary of polytomous items), but the performance of summary intervals for those approaches was not studied. Future directions include examining summary intervals for model-based CA and CC with discrete items. In conclusion, researchers are encouraged to report CA and CC indices, along with bootstrap confidence intervals, whenever they are using scores to make decisions about respondents.

## Supplemental Material

sj-docx-1-epm-10.1177_00131644221092347 – Supplemental material for Summary Intervals for Model-Based Classification Accuracy and Consistency Indices Click here for additional data file. Supplemental material, sj-docx-1-epm-10.1177_00131644221092347 for Summary Intervals for Model-Based Classification Accuracy and Consistency Indices by Oscar Gonzalez in Educational and Psychological Measurement

## Notes

1. The term “summary intervals” is used throughout this article to refer jointly to bootstrap confidence intervals and Bayesian credible intervals.

2. Note that these priors encode information from an analysis using the same data, which are referred to in the literature as data-dependent priors (van de Schoot et al., 2021). These priors were used for illustrative purposes because they are easy to automate in a simulation, but the use of these priors in applied settings is discouraged because of double-dipping (i.e., using the data to determine the prior and posterior distribution). In this article, the issue was mitigated by adding more uncertainty to prior as what the maximum likelihood solution suggested. As such, we could treat these priors as empirical, weakly informative priors.

3. Because the relation of the summed score and η is bivariate normal, using cutpoints at −0.75 *SD* and −1.5 *SD* would yield the same estimates of classification accuracy and consistency as studying the relations at 0.75 *SD* and 1.5 *SD*, but the values of sensitivity and specificity would swap.

## References

[bibr1-00131644221092347] American Educational Research Association, American Psychological Association, & National Council on Measurement in Education. (2014). Standards for educational and psychological testing. American Educational Research Association.

[bibr2-00131644221092347] Brooks S. P. Gelman A. (1998). Convergence assessment techniques for Markov chain Monte Carlo. Statistics and Computing, 8, 319–335.

[bibr3-00131644221092347] Brown K. W. Ryan R. M. (2003). The benefits of being present: Mindfulness and its role in psychological well-being. Journal of Personality and Social Psychology, 84, 822–848. 12703651 10.1037/0022-3514.84.4.822

[bibr4-00131644221092347] Casella G. George E. I. (1992). Explaining the Gibbs sampler. The American Statistician, 46, 167–174.

[bibr5-00131644221092347] Edwards M. C. (2010). A Markov chain Monte Carlo approach to confirmatory item factor analysis. Psychometrika, 75, 474–497.

[bibr6-00131644221092347] Edwards M. C. Houts C. R. Cai L. (2018). A diagnostic procedure to detect departures from local independence in item response theory models. Psychological Methods, 23, 138–149. 28368176 10.1037/met0000121 PMC5624819

[bibr7-00131644221092347] Efron B. Tibshirani R. J. (1993). An introduction to the bootstrap. CRC Press.

[bibr8-00131644221092347] Eisenberg I. W. Bissett P. G. Canning J. R. Dallery J. Enkavi A. Z. Gabrieli S. W. Gonzalez O. Green A. I. Greene M. A. Kiernan M. Kim S. J. Li J. Lowe M. Mazza G. L. Metcalf S. A. Onken L. Peters E. Prochaska J. J. Scherer E. A. . . .Poldrack R. A. (2018). Applying novel technologies and methods to inform the ontology of self-regulation. Behavior Research and Therapy, 101, 46–57. 10.1016/j.brat.2017.09.014 PMC5801197 29066077

[bibr9-00131644221092347] Gelman A. Carlin J. B. Stern H. S. Rubin D. B. (2004). Bayesian data analysis. CRC Press.

[bibr10-00131644221092347] Gonzalez O. Georgeson A. R. Pelham W. E. III. Fouladi R. T. (2021). Estimating classification consistency of screening measures and quantifying the impact of measurement bias. Psychological Assessment, 37, 596–609. 10.1037/pas0000938 PMC8412438 33998821

[bibr11-00131644221092347] Gonzalez O. Pelham W. E. III . (2021). When does differential item functioning matter for screening? A method for empirical evaluation. Assessment, 28, 446–456. 32248701 10.1177/1073191120913618 PMC9705193

[bibr12-00131644221092347] Kelley K. Pornprasertmanit S. (2016). Confidence intervals for population reliability coefficients: Evaluation of methods, recommendations, and software for composite measures. Psychological Methods, 21, 69–92. 26962759 10.1037/a0040086

[bibr13-00131644221092347] Lee W. C. (2010). Classification consistency and accuracy for complex assessments using item response theory. Journal of Educational Measurement, 47, 1–17.

[bibr14-00131644221092347] Levy R. Mislevy R. J. (2017). Bayesian psychometric modeling. CRC Press.

[bibr15-00131644221092347] Livingston S. A. Lewis C. (1995). Estimating the consistency and accuracy of classifications based on test scores. Journal of Educational Measurement, 32, 179–197.

[bibr16-00131644221092347] MacCallum R. C. Widaman K. F. Preacher K. J. Hong S. (2001). Sample size in factor analysis: The role of model error. Multivariate Behavioral Research, 36, 611–637. 26822184 10.1207/S15327906MBR3604_06

[bibr17-00131644221092347] McDonald R.P. (1999). Test theory: A unified treatment. Lawrence Erlbaum.

[bibr18-00131644221092347] Millsap R. E. Kwok O. M. (2004). Evaluating the impact of partial factorial invariance on selection in two populations. Psychological Methods, 9, 93–115. 15053721 10.1037/1082-989X.9.1.93

[bibr19-00131644221092347] Moses T. Kim S. (2015). Methods for evaluating composite reliability, classification consistency, and classification accuracy for mixed-format licensure tests. Applied Psychological Measurement, 39, 314–329. 29881011 10.1177/0146621614563067 PMC5978540

[bibr20-00131644221092347] Newcombe R. G. (1998). Two-sided confidence intervals for the single proportion: Comparison of seven methods. Statistics in Medicine, 17, 857–872. 9595616 10.1002/(sici)1097-0258(19980430)17:8<857::aid-sim777>3.0.co;2-e

[bibr21-00131644221092347] Pepe M. S. (2003). The statistical evaluation of medical tests for classification and prediction. Oxford University Press.

[bibr22-00131644221092347] Pfadt J. M. van den Bergh D. Sijtsma K. Moshagen M. Wagenmakers E. J. (2021). Bayesian estimation of single-test reliability coefficients. Multivariate Behavioral Research. Advance online publication. 10.1080/00273171.2021.1891855 33759671

[bibr23-00131644221092347] Plummer M. (2003). JAGS: A program for analysis of Bayesian graphical models using Gibbs sampling. In Hornik K. Leisch F. Zeileis A. (Eds.), Proceedings of the 3rd International Workshop on Distributed Statistical Computing. http://www.ci.tuwien.ac.at/Conferences/DSC-2003/

[bibr24-00131644221092347] Plummer M. Best N. Cowles K. Vines K. (2006). CODA: Convergence diagnosis and output analysis for MCMC. R News, 6, 7–11.

[bibr25-00131644221092347] Reise S. P. Scheines R. Widaman K. F. Haviland M. G. (2013). Multidimensionality and structural coefficient bias in structural equation modeling: A bifactor perspective. Educational and Psychological Measurement, 73, 5–26.

[bibr26-00131644221092347] Rosseel Y. (2012). lavaan: An R package for structural equation modeling. Journal of Statistical Software, 48, 1–36.

[bibr27-00131644221092347] Su Y.-S. Yajima M. (2020). R2jags: Using R to run “JAGS” (R Package Version 0.6-1). https://CRAN.R-project.org/package=R2jags

[bibr28-00131644221092347] Tanzer J. R. Harlow L. L. (2021). Bayesian modeling of test reliability. Multivariate Behavioral Research, 56, 159–159. 33275469 10.1080/00273171.2020.1854082

[bibr29-00131644221092347] van de Schoot R. Depaoli S. King R. Kramer B. Märtens K. Tadesse M. G. Vannucci M. Gelman A. Veen D. Willemsen J. Yau C. (2021). Bayesian statistics and modelling. Nature Reviews Methods Primers, 1, 1–26.

[bibr30-00131644221092347] van Erp S. Mulder J. Oberski D. L. (2018). Prior sensitivity analysis in default Bayesian structural equation modeling. Psychological Methods, 23, 363–388. 29172613 10.1037/met0000162

[bibr31-00131644221092347] Veen D. Stoel D. Zondervan-Zwijnenburg M. Van de Schoot R. (2017). Proposal for a five-step method to elicit expert judgment. Frontiers in Psychology, 8, Article 2110. 10.3389/fpsyg.2017.02110 PMC5723340 29259569

[bibr32-00131644221092347] Youngstrom E. A. (2014). A primer on receiver operating characteristic analysis and diagnostic efficiency statistics for pediatric psychology: We are ready to ROC. Journal of Pediatric Psychology, 39, 204–221. 23965298 10.1093/jpepsy/jst062 PMC3936258

