# Supplementary material for: Summary Intervals for Model-Based Classification Accuracy and Consistency Indices
Source: Educ Psychol Meas. 2022 Apr 28;83(2):240–61. doi: 10.1177/00131644221092347 (PMC9972125; doi:10.1177/00131644221092347)
Supplement: sj-docx-1-epm-10.1177_00131644221092347 – Supplemental material for Summary Intervals for Model-Based Classification Accuracy and Consistency Indices [file sj-docx-1-epm-10.1177_00131644221092347.docx]

**Supplementary materials**

| Table A0. *True factor loadings for simulation conditions* | | | | | |
| --- | --- | --- | --- | --- | --- |
| Average factor loading | | | | | |
| f = .5 | | f = .6 | | f = .7 | |
| .635 | .376 | .735 | .476 | .835 | .576 |
| .551 | .672 | .651 | .772 | .751 | .872 |
| .626 | .360 | .726 | .460 | .826 | .560 |
| .613 | .381 | .713 | .481 | .813 | .581 |
| .488 | .417 | .588 | .517 | .688 | .617 |
| .452 | .334 | .552 | .434 | .652 | .534 |
| .312 | .320 | .412 | .420 | .512 | .620 |
| .595 | .395 | .695 | .495 | .795 | .595 |
| .386 | .366 | .486 | .466 | .586 | .566 |
| .604 | .551 | .704 | .651 | .804 | .751 |
| Note: values in dark grey cells are for conditions with 5 items, values in dark grey and light grey cells are for conditions with 10 items, and values in dark grey, light grey, and white cells are for conditions with 20 items. | | | | | |

| Table A1. *True values for the classification accuracy and consistency indices* | | | | | | |
| --- | --- | --- | --- | --- | --- | --- |
|  | Average factor loading | | | | | |
|  | .5 | .6 | .7 | .5 | .6 | .7 |
|  | Cutpoint at Mean | | | | | |
| *# items* | Classification rate | | | Sensitivity | | |
| 5 | .823 | .859 | .892 | .823 | .859 | .892 |
| 10 | .851 | .882 | .909 | .851 | .882 | .909 |
| 20 | .875 | .902 | .924 | .875 | .902 | .924 |
|  | Specificity | | | Consistency | | |
| 5 | .823 | .859 | .892 | .756 | .804 | .849 |
| 10 | .851 | .882 | .909 | .793 | .835 | .872 |
| 20 | .875 | .902 | .924 | .826 | .863 | .894 |
|  | Cutpoint at .75SD | | | | | |
|  | Classification rate | | | Sensitivity | | |
| 5 | .867 | .894 | .919 | .707 | .766 | .821 |
| 10 | .888 | .911 | .931 | .753 | .804 | .848 |
| 20 | .906 | .926 | .946 | .793 | .837 | .874 |
|  | Specificity | | | Consistency | | |
| 5 | .914 | .931 | .947 | .819 | .853 | .886 |
| 10 | .927 | .943 | .955 | .845 | .876 | .903 |
| 20 | .939 | .952 | .963 | .869 | .897 | .920 |
|  | Cutpoint at 1.5SD | | | | | |
|  | Classification rate | | | Sensitivity | | |
| 5 | .944 | .955 | .965 | .582 | .663 | .740 |
| 10 | .953 | .962 | .971 | .645 | .717 | .780 |
| 20 | .960 | .968 | .976 | .701 | .764 | .817 |
|  | Specificity | | | Consistency | | |
| 5 | .970 | .976 | .981 | .925 | .939 | .952 |
| 10 | .975 | .980 | .984 | .935 | .948 | .959 |
| 20 | .979 | .983 | .987 | .945 | .956 | .966 |

Table A2. *Relative bias and width of the bootstrap confidence intervals with cutpoint at the mean*

|  |  | Relative bias | | | 95% equal-tail interval width | | | 95% HPD interval width | | |
| --- | --- | --- | --- | --- | --- | --- | --- | --- | --- | --- |
| i | n | *f* = .5 | *f* = .6 | *f* = .7 | *f* = .5 | *f* = .6 | *f* = .7 | *f* = .5 | *f* = .6 | *f* = .7 |
|  |  | *Classification Rate* | | | | | | | | |
| 5 | 200 | -0.003 | -0.004 | -0.005 | 0.045 | 0.037 | 0.032 | 0.044 | 0.036 | 0.031 |
|  | 500 | -0.004 | -0.005 | -0.005 | 0.037 | 0.032 | 0.031 | 0.036 | 0.031 | 0.029 |
| 10 | 200 | -0.004 | -0.005 | -0.006 | 0.032 | 0.030 | 0.032 | 0.032 | 0.029 | 0.030 |
|  | 500 | -0.001 | -0.002 | -0.002 | 0.028 | 0.022 | 0.018 | 0.027 | 0.022 | 0.017 |
| 20 | 200 | -0.002 | -0.002 | -0.003 | 0.022 | 0.018 | 0.016 | 0.022 | 0.018 | 0.016 |
|  | 500 | -0.002 | -0.002 | -0.002 | 0.018 | 0.016 | 0.015 | 0.018 | 0.016 | 0.015 |
|  |  | *Sensitivity* | | | | | | | | |
| 5 | 200 | -0.002 | -0.004 | -0.003 | 0.118 | 0.115 | 0.112 | 0.116 | 0.113 | 0.110 |
|  | 500 | -0.003 | -0.002 | -0.003 | 0.115 | 0.112 | 0.112 | 0.113 | 0.109 | 0.109 |
| 10 | 200 | -0.004 | -0.002 | -0.003 | 0.114 | 0.111 | 0.110 | 0.112 | 0.108 | 0.107 |
|  | 500 | 0.000 | -0.002 | -0.001 | 0.074 | 0.073 | 0.071 | 0.073 | 0.072 | 0.070 |
| 20 | 200 | -0.002 | 0.000 | -0.003 | 0.073 | 0.071 | 0.071 | 0.072 | 0.070 | 0.070 |
|  | 500 | -0.001 | -0.001 | -0.002 | 0.071 | 0.071 | 0.070 | 0.070 | 0.070 | 0.069 |
|  |  | *Specificity* | | | | | | | | |
| 5 | 200 | -0.002 | -0.002 | -0.003 | 0.118 | 0.114 | 0.112 | 0.116 | 0.112 | 0.110 |
|  | 500 | -0.002 | -0.005 | -0.004 | 0.115 | 0.114 | 0.112 | 0.113 | 0.112 | 0.109 |
| 10 | 200 | -0.001 | -0.004 | -0.005 | 0.113 | 0.112 | 0.112 | 0.111 | 0.110 | 0.108 |
|  | 500 | -0.002 | -0.002 | -0.001 | 0.075 | 0.073 | 0.071 | 0.074 | 0.072 | 0.070 |
| 20 | 200 | -0.001 | -0.003 | -0.001 | 0.072 | 0.072 | 0.070 | 0.071 | 0.071 | 0.069 |
|  | 500 | -0.001 | -0.001 | -0.001 | 0.071 | 0.070 | 0.070 | 0.070 | 0.069 | 0.069 |
|  |  | *Classification Consistency* | | | | | | | | |
| 5 | 200 | 0.002 | 0.001 | 0.000 | 0.055 | 0.044 | 0.033 | 0.055 | 0.043 | 0.033 |
|  | 500 | 0.001 | 0.000 | 0.000 | 0.044 | 0.035 | 0.027 | 0.043 | 0.034 | 0.026 |
| 10 | 200 | 0.001 | 0.000 | -0.001 | 0.036 | 0.028 | 0.022 | 0.035 | 0.027 | 0.021 |
|  | 500 | 0.001 | 0.000 | 0.000 | 0.035 | 0.028 | 0.021 | 0.035 | 0.027 | 0.021 |
| 20 | 200 | 0.000 | 0.000 | 0.000 | 0.028 | 0.022 | 0.017 | 0.028 | 0.022 | 0.017 |
|  | 500 | 0.000 | 0.000 | -0.001 | 0.022 | 0.018 | 0.014 | 0.022 | 0.017 | 0.013 |

**Note***:* i is for number of items, n is for sample size, and f is for average factor loading

Table A3. *Bootstrap confidence interval coverage and balance [lower, upper] for a cutpoint at .75SD*

|  |  | 95% Equal-tail bootstrap confidence interval | | | 95% HPD Bootstrap confidence interval | | |
| --- | --- | --- | --- | --- | --- | --- | --- |
| I | n | *f* = .5 | *f* = .6 | *f* = .7 | *f* = .5 | *f* = .6 | *f* = .7 |
|  |  | *Classification Rate* | | | | | |
| 5 | 200 | 0.959[0.008,0.033] | 0.946[0.000,0.054] | 0.934[0.000,0.066] | 0.960[0.013,0.027] | 0.954[0.001,0.045] | 0.961[0.000,0.039] |
|  | 500 | 0.943[0.003,0.054] | 0.918[0.000,0.082] | 0.926[0.000,0.074] | 0.954[0.007,0.039] | 0.948[0.000,0.052] | 0.959[0.000,0.041] |
| 10 | 200 | 0.947[0.000,0.053] | 0.939[0.000,0.061] | **0.886[0.000,0.114]** | 0.962[0.000,0.038] | 0.963[0.000,0.037] | 0.939[0.000,0.061] |
|  | 500 | 0.968[0.012,0.020] | 0.960[0.011,0.029] | 0.943[0.001,0.056] | 0.967[0.015,0.018] | 0.961[0.012,0.027] | 0.953[0.001,0.046] |
| 20 | 200 | 0.967[0.003,0.030] | 0.965[0.000,0.035] | 0.967[0.000,0.033] | 0.964[0.005,0.031] | 0.967[0.002,0.031] | **0.979[0.000,0.021]** |
|  | 500 | 0.962[0.000,0.038] | 0.969[0.000,0.031] | 0.930[0.000,0.070] | 0.968[0.000,0.032] | 0.974[0.001,0.025] | 0.959[0.000,0.041] |
|  |  | *Sensitivity* | | | | | |
| 5 | 200 | 0.946[0.019,0.035] | 0.934[0.023,0.043] | 0.936[0.011,0.053] | 0.939[0.028,0.033] | 0.930[0.028,0.042] | 0.926[0.027,0.047] |
|  | 500 | 0.939[0.020,0.041] | 0.935[0.018,0.047] | 0.937[0.017,0.046] | 0.932[0.027,0.041] | 0.926[0.032,0.042] | 0.927[0.030,0.043] |
| 10 | 200 | **0.919[0.024,0.057]** | 0.950[0.020,0.030] | 0.934[0.018,0.048] | **0.920[0.033,0.047]** | 0.948[0.025,0.027] | **0.922[0.040,0.038]** |
|  | 500 | 0.937[0.022,0.041] | 0.955[0.015,0.030] | 0.930[0.023,0.047] | 0.936[0.029,0.035] | 0.946[0.023,0.031] | 0.929[0.031,0.040] |
| 20 | 200 | 0.947[0.013,0.040] | 0.938[0.024,0.038] | 0.951[0.018,0.031] | 0.943[0.017,0.040] | 0.929[0.033,0.038] | 0.946[0.029,0.025] |
|  | 500 | 0.950[0.018,0.032] | 0.948[0.021,0.031] | 0.935[0.020,0.045] | 0.945[0.026,0.029] | 0.945[0.028,0.027] | **0.923[0.038,0.039]** |
|  |  | *Specificity* | | | | | |
| 5 | 200 | 0.957[0.026,0.017] | 0.936[0.040,0.024] | 0.940[0.042,0.018] | 0.943[0.040,0.017] | **0.918[0.059,0.023]** | 0.925[0.062,0.013] |
|  | 500 | 0.947[0.034,0.019] | 0.946[0.038,0.016] | 0.946[0.032,0.022] | 0.936[0.050,0.014] | 0.939[0.049,0.012] | 0.930[0.055,0.015] |
| 10 | 200 | 0.937[0.046,0.017] | 0.950[0.031,0.019] | 0.933[0.045,0.022] | 0.926[0.060,0.014] | 0.939[0.044,0.017] | **0.921[0.068,0.011]** |
|  | 500 | 0.949[0.030,0.021] | 0.951[0.034,0.015] | 0.940[0.041,0.019] | 0.947[0.035,0.018] | 0.949[0.039,0.012] | 0.935[0.048,0.017] |
| 20 | 200 | 0.947[0.039,0.014] | 0.936[0.032,0.032] | 0.950[0.033,0.017] | 0.946[0.041,0.013] | 0.934[0.038,0.028] | 0.936[0.050,0.014] |
|  | 500 | 0.961[0.026,0.013] | 0.950[0.033,0.017] | 0.949[0.032,0.019] | 0.946[0.040,0.014] | 0.941[0.044,0.015] | 0.934[0.052,0.014] |
|  |  | *Classification Consistency* | | | | | |
| 5 | 200 | 0.939[0.041,0.020] | 0.944[0.047,0.009] | 0.941[0.046,0.013] | 0.943[0.040,0.017] | 0.943[0.048,0.009] | 0.940[0.050,0.010] |
|  | 500 | 0.944[0.041,0.015] | 0.958[0.026,0.016] | 0.950[0.035,0.015] | 0.935[0.049,0.016] | 0.957[0.028,0.015] | 0.941[0.045,0.014] |
| 10 | 200 | 0.949[0.035,0.016] | 0.946[0.038,0.016] | 0.958[0.019,0.023] | 0.942[0.043,0.015] | 0.937[0.046,0.017] | 0.952[0.025,0.023] |
|  | 500 | 0.949[0.030,0.021] | 0.954[0.030,0.016] | 0.963[0.028,0.009] | 0.950[0.029,0.021] | 0.952[0.034,0.014] | 0.958[0.030,0.012] |
| 20 | 200 | 0.962[0.027,0.011] | 0.953[0.028,0.019] | 0.955[0.039,0.006] | 0.949[0.038,0.013] | 0.945[0.034,0.021] | 0.951[0.042,0.007] |
|  | 500 | 0.964[0.026,0.010] | 0.953[0.029,0.018] | 0.967[0.016,0.017] | 0.957[0.034,0.009] | 0.951[0.033,0.016] | 0.962[0.017,0.021] |

**Note***:* i is for number of items, n is for sample size, and f is for average factor loading, and in bold are intervals outside the robustness criterion

Table A4. *Relative bias and width of the bootstrap confidence intervals for cutpoint at .75SD*

|  |  | Relative bias | | | 95% equal-tail interval width | | | 95% HPD interval width | | |
| --- | --- | --- | --- | --- | --- | --- | --- | --- | --- | --- |
| i | n | *f* = .5 | *f* = .6 | *f* = .7 | *f* = .5 | *f* = .6 | *f* = .7 | *f* = .5 | *f* = .6 | *f* = .7 |
|  |  | *Classification Rate* | | | | | | | | |
| 5 | 200 | -0.002 | -0.003 | -0.004 | 0.031 | 0.027 | 0.025 | 0.030 | 0.026 | 0.024 |
|  | 500 | -0.003 | -0.004 | -0.004 | 0.026 | 0.024 | 0.025 | 0.026 | 0.024 | 0.024 |
| 10 | 200 | -0.003 | -0.004 | -0.005 | 0.024 | 0.024 | 0.028 | 0.024 | 0.023 | 0.026 |
|  | 500 | -0.001 | -0.002 | -0.002 | 0.019 | 0.015 | 0.013 | 0.019 | 0.015 | 0.013 |
| 20 | 200 | -0.001 | -0.002 | -0.002 | 0.015 | 0.013 | 0.013 | 0.015 | 0.013 | 0.012 |
|  | 500 | -0.001 | -0.001 | -0.002 | 0.013 | 0.012 | 0.013 | 0.013 | 0.012 | 0.012 |
|  |  | *Sensitivity* | | | | | | | | |
| 5 | 200 | -0.009 | -0.011 | -0.010 | 0.209 | 0.205 | 0.205 | 0.206 | 0.202 | 0.200 |
|  | 500 | -0.011 | -0.010 | -0.010 | 0.205 | 0.203 | 0.204 | 0.202 | 0.199 | 0.198 |
| 10 | 200 | -0.011 | -0.009 | -0.013 | 0.205 | 0.203 | 0.203 | 0.201 | 0.197 | 0.196 |
|  | 500 | -0.001 | -0.004 | -0.003 | 0.133 | 0.131 | 0.130 | 0.131 | 0.129 | 0.128 |
| 20 | 200 | -0.006 | -0.001 | -0.005 | 0.130 | 0.129 | 0.130 | 0.129 | 0.127 | 0.127 |
|  | 500 | -0.005 | -0.004 | -0.006 | 0.130 | 0.130 | 0.130 | 0.128 | 0.127 | 0.127 |
|  |  | *Specificity* | | | | | | | | |
| 5 | 200 | 0.001 | 0.001 | 0.000 | 0.065 | 0.063 | 0.062 | 0.063 | 0.062 | 0.061 |
|  | 500 | 0.001 | -0.001 | 0.000 | 0.063 | 0.062 | 0.062 | 0.062 | 0.061 | 0.060 |
| 10 | 200 | 0.001 | 0.000 | -0.001 | 0.062 | 0.062 | 0.061 | 0.061 | 0.060 | 0.059 |
|  | 500 | 0.000 | 0.000 | 0.000 | 0.041 | 0.040 | 0.040 | 0.041 | 0.040 | 0.039 |
| 20 | 200 | 0.001 | -0.001 | 0.001 | 0.040 | 0.040 | 0.039 | 0.039 | 0.039 | 0.038 |
|  | 500 | 0.000 | 0.000 | 0.000 | 0.040 | 0.039 | 0.039 | 0.039 | 0.038 | 0.038 |
|  |  | *Classification Consistency* | | | | | | | | |
| 5 | 200 | 0.003 | 0.003 | 0.002 | 0.046 | 0.037 | 0.028 | 0.046 | 0.037 | 0.028 |
|  | 500 | 0.003 | 0.001 | 0.002 | 0.037 | 0.030 | 0.023 | 0.037 | 0.030 | 0.023 |
| 10 | 200 | 0.003 | 0.001 | 0.000 | 0.031 | 0.024 | 0.019 | 0.030 | 0.024 | 0.019 |
|  | 500 | 0.001 | 0.001 | 0.001 | 0.029 | 0.023 | 0.018 | 0.029 | 0.023 | 0.018 |
| 20 | 200 | 0.001 | 0.000 | 0.001 | 0.023 | 0.019 | 0.014 | 0.023 | 0.018 | 0.014 |
|  | 500 | 0.001 | 0.000 | 0.000 | 0.019 | 0.015 | 0.012 | 0.019 | 0.015 | 0.012 |

**Note***:* i is for number of items, n is for sample size, and f is for average factor loading

Table A5. *Bootstrap confidence interval coverage and balance [lower, upper] for a cutpoint at 1.5SD*

|  |  | 95% Equal-tail bootstrap confidence interval | | | 95% HPD Bootstrap confidence interval | | |
| --- | --- | --- | --- | --- | --- | --- | --- |
| I | n | *f* = .5 | *f* = .6 | *f* = .7 | *f* = .5 | *f* = .6 | *f* = .7 |
|  |  | *Classification Rate* | | | | | |
| 5 | 200 | 0.970[0.010,0.020] | **0.978[0.000,0.022]** | **0.981[0.000,0.019]** | 0.973[0.015,0.012] | **0.988[0.000,0.012]** | **0.992[0.000,0.008]** |
|  | 500 | 0.964[0.000,0.036] | 0.969[0.000,0.031] | 0.925[0.000,0.075] | **0.978[0.001,0.021]** | **0.988[0.000,0.012]** | 0.968[0.000,0.032] |
| 10 | 200 | 0.973[0.000,0.027] | **0.977[0.000,0.023]** | **0.874[0.000,0.126]** | **0.988[0.000,0.012]** | **0.995[0.000,0.005]** | 0.951[0.000,0.049] |
|  | 500 | 0.974[0.011,0.015] | **0.982[0.003,0.015]** | **0.989[0.000,0.011]** | **0.980[0.014,0.006]** | **0.986[0.004,0.010]** | **0.995[0.000,0.005]** |
| 20 | 200 | 0.973[0.001,0.026] | **0.982[0.000,0.018]** | 0.946[0.000,0.054] | **0.982[0.001,0.017]** | **0.996[0.000,0.004]** | **0.982[0.000,0.018]** |
|  | 500 | **0.985[0.000,0.015]** | **0.995[0.000,0.005]** | **0.921[0.000,0.079]** | **0.994[0.000,0.006]** | **0.997[0.000,0.003]** | **0.974[0.000,0.026]** |
|  |  | *Sensitivity* | | | | | |
| 5 | 200 | 0.930[0.021,0.049] | 0.934[0.018,0.048] | 0.933[0.014,0.053] | **0.921[0.026,0.053]** | **0.921[0.029,0.050]** | **0.924[0.023,0.053]** |
|  | 500 | 0.946[0.013,0.041] | **0.924[0.014,0.062]** | 0.926[0.018,0.056] | 0.930[0.023,0.047] | **0.911[0.030,0.059]** | **0.915[0.031,0.054]** |
| 10 | 200 | **0.923[0.018,0.059]** | 0.945[0.015,0.040] | 0.934[0.017,0.049] | **0.909[0.031,0.060]** | 0.936[0.028,0.036] | 0.928[0.030,0.042] |
|  | 500 | 0.940[0.022,0.038] | 0.942[0.018,0.040] | 0.936[0.024,0.040] | 0.934[0.027,0.039] | 0.943[0.022,0.035] | 0.925[0.035,0.040] |
| 20 | 200 | 0.945[0.012,0.043] | 0.947[0.016,0.037] | 0.939[0.022,0.039] | 0.942[0.017,0.041] | 0.931[0.030,0.039] | 0.937[0.028,0.035] |
|  | 500 | 0.941[0.020,0.039] | 0.947[0.018,0.035] | 0.930[0.024,0.046] | 0.936[0.028,0.036] | 0.941[0.028,0.031] | **0.914[0.038,0.048]** |
|  |  | *Specificity* | | | | | |
| 5 | 200 | 0.945[0.041,0.014] | 0.932[0.045,0.023] | 0.933[0.057,0.010] | 0.930[0.062,0.008] | 0.925[0.060,0.015] | **0.916[0.078,0.006]** |
|  | 500 | 0.950[0.037,0.013] | 0.939[0.046,0.015] | 0.940[0.048,0.012] | 0.932[0.055,0.013] | **0.923[0.068,0.009]** | **0.913[0.078,0.009]** |
| 10 | 200 | 0.926[0.054,0.020] | 0.949[0.035,0.016] | 0.944[0.044,0.012] | **0.914[0.073,0.013**] | 0.925[0.062,0.013] | **0.916[0.076,0.008]** |
|  | 500 | 0.947[0.040,0.013] | 0.955[0.029,0.016] | 0.943[0.044,0.013] | 0.936[0.052,0.012] | 0.946[0.043,0.011] | 0.934[0.055,0.011] |
| 20 | 200 | 0.955[0.033,0.012] | 0.951[0.028,0.021] | 0.950[0.036,0.014] | 0.946[0.044,0.010] | 0.935[0.047,0.018] | 0.936[0.052,0.012] |
|  | 500 | 0.958[0.024,0.018] | 0.955[0.033,0.012] | 0.947[0.038,0.015] | 0.951[0.035,0.014] | 0.947[0.045,0.008] | 0.930[0.058,0.012] |
|  |  | *Classification Consistency* | | | | | |
| 5 | 200 | 0.941[0.046,0.013] | 0.937[0.041,0.022] | 0.942[0.048,0.010] | 0.940[0.049,0.011] | 0.934[0.043,0.023] | 0.937[0.054,0.009] |
|  | 500 | 0.947[0.047,0.006] | 0.942[0.045,0.013] | 0.939[0.047,0.014] | 0.936[0.055,0.009] | 0.940[0.047,0.013] | 0.938[0.047,0.015] |
| 10 | 200 | 0.928[0.055,0.017] | 0.945[0.040,0.015] | 0.945[0.040,0.015] | **0.924[0.059,0.017]** | 0.943[0.041,0.016] | 0.943[0.041,0.016] |
|  | 500 | 0.949[0.040,0.011] | 0.957[0.025,0.018] | 0.957[0.032,0.011] | 0.941[0.047,0.012] | 0.958[0.024,0.018] | 0.955[0.033,0.012] |
| 20 | 200 | 0.944[0.048,0.008] | 0.945[0.034,0.021] | 0.961[0.026,0.013] | 0.939[0.053,0.008] | 0.945[0.035,0.020] | 0.954[0.031,0.015] |
|  | 500 | 0.958[0.032,0.010] | 0.957[0.031,0.012] | 0.959[0.027,0.014] | 0.960[0.028,0.012] | 0.954[0.034,0.012] | 0.956[0.027,0.017] |

**Note***:* i is for number of items, n is for sample size, and f is for average factor loading, and in bold are intervals outside the robustness criterion

Table A6. *Relative bias and width of the bootstrap confidence intervals for cutpoint at 1.5SD*

|  |  | Relative bias | | | 95% equal-tail interval width | | | 95% HPD interval width | | |
| --- | --- | --- | --- | --- | --- | --- | --- | --- | --- | --- |
| i | n | *f* = .5 | *f* = .6 | *f* = .7 | *f* = .5 | *f* = .6 | *f* = .7 | *f* = .5 | *f* = .6 | *f* = .7 |
|  |  | *Classification Rate* | | | | | | | | |
| 5 | 200 | 0.000 | -0.001 | -0.002 | 0.015 | 0.014 | 0.014 | 0.014 | 0.013 | 0.013 |
|  | 500 | -0.002 | -0.002 | -0.003 | 0.013 | 0.014 | 0.015 | 0.013 | 0.012 | 0.014 |
| 10 | 200 | -0.002 | -0.002 | -0.003 | 0.013 | 0.014 | 0.017 | 0.012 | 0.013 | 0.015 |
|  | 500 | 0.000 | -0.001 | -0.001 | 0.009 | 0.008 | 0.007 | 0.009 | 0.007 | 0.007 |
| 20 | 200 | -0.001 | -0.001 | -0.001 | 0.007 | 0.007 | 0.007 | 0.007 | 0.007 | 0.007 |
|  | 500 | -0.001 | 0.000 | -0.002 | 0.007 | 0.007 | 0.008 | 0.007 | 0.006 | 0.007 |
|  |  | *Sensitivity* | | | | | | | | |
| 5 | 200 | -0.020 | -0.021 | -0.022 | 0.316 | 0.323 | 0.335 | 0.313 | 0.319 | 0.327 |
|  | 500 | -0.023 | -0.026 | -0.025 | 0.322 | 0.330 | 0.338 | 0.318 | 0.323 | 0.328 |
| 10 | 200 | -0.023 | -0.023 | -0.030 | 0.329 | 0.335 | 0.341 | 0.323 | 0.326 | 0.328 |
|  | 500 | -0.004 | -0.009 | -0.005 | 0.205 | 0.210 | 0.216 | 0.202 | 0.208 | 0.213 |
| 20 | 200 | -0.011 | -0.005 | -0.010 | 0.208 | 0.214 | 0.220 | 0.206 | 0.211 | 0.216 |
|  | 500 | -0.009 | -0.009 | -0.012 | 0.213 | 0.219 | 0.223 | 0.210 | 0.216 | 0.218 |
|  |  | *Specificity* | | | | | | | | |
| 5 | 200 | 0.001 | 0.000 | 0.000 | 0.032 | 0.031 | 0.031 | 0.032 | 0.030 | 0.029 |
|  | 500 | 0.000 | 0.000 | 0.000 | 0.031 | 0.031 | 0.030 | 0.030 | 0.029 | 0.029 |
| 10 | 200 | 0.000 | 0.000 | -0.001 | 0.031 | 0.030 | 0.030 | 0.030 | 0.029 | 0.028 |
|  | 500 | 0.000 | 0.000 | 0.000 | 0.021 | 0.020 | 0.020 | 0.021 | 0.020 | 0.019 |
| 20 | 200 | 0.000 | -0.001 | 0.000 | 0.020 | 0.020 | 0.019 | 0.020 | 0.019 | 0.019 |
|  | 500 | 0.000 | 0.000 | 0.000 | 0.020 | 0.019 | 0.019 | 0.019 | 0.019 | 0.018 |
|  |  | *Classification Consistency* | | | | | | | | |
| 5 | 200 | 0.003 | 0.001 | 0.001 | 0.038 | 0.031 | 0.024 | 0.038 | 0.030 | 0.023 |
|  | 500 | 0.002 | 0.001 | 0.001 | 0.032 | 0.026 | 0.020 | 0.032 | 0.025 | 0.020 |
| 10 | 200 | 0.002 | 0.001 | 0.000 | 0.027 | 0.021 | 0.016 | 0.027 | 0.021 | 0.016 |
|  | 500 | 0.001 | 0.000 | 0.000 | 0.024 | 0.020 | 0.015 | 0.024 | 0.019 | 0.015 |
| 20 | 200 | 0.001 | 0.000 | 0.000 | 0.020 | 0.016 | 0.013 | 0.020 | 0.016 | 0.013 |
|  | 500 | 0.001 | 0.001 | 0.000 | 0.017 | 0.013 | 0.011 | 0.017 | 0.013 | 0.010 |

**Note***:* i is for number of items, n is for sample size, and f is for average factor loading

Table B1. *Relative bias and width of the Bayesian credible intervals (diffused priors) with cutpoint at mean*

|  |  | Relative bias | | | 95% equal-tail interval width | | | 95% HPD interval width | | |
| --- | --- | --- | --- | --- | --- | --- | --- | --- | --- | --- |
| i | n | *f* = .5 | *f* = .6 | *f* = .7 | *f* = .5 | *f* = .6 | *f* = .7 | *f* = .5 | *f* = .6 | *f* = .7 |
|  |  | *Classification Rate* | | | | | | | | |
| 5 | 200 | -0.038 | -0.035 | -0.035 | 0.054 | 0.043 | 0.036 | 0.054 | 0.043 | 0.035 |
|  | 500 | -0.023 | -0.022 | -0.022 | 0.041 | 0.034 | 0.031 | 0.041 | 0.034 | 0.030 |
| 10 | 200 | -0.012 | -0.012 | -0.014 | 0.034 | 0.031 | 0.031 | 0.034 | 0.030 | 0.030 |
|  | 500 | -0.016 | -0.016 | -0.015 | 0.030 | 0.024 | 0.019 | 0.030 | 0.024 | 0.019 |
| 20 | 200 | -0.010 | -0.009 | -0.010 | 0.023 | 0.019 | 0.016 | 0.023 | 0.019 | 0.016 |
|  | 500 | -0.006 | -0.005 | -0.006 | 0.019 | 0.016 | 0.016 | 0.019 | 0.016 | 0.015 |
|  |  | *Sensitivity* | | | | | | | | |
| 5 | 200 | -0.037 | -0.035 | -0.034 | 0.122 | 0.117 | 0.114 | 0.121 | 0.116 | 0.113 |
|  | 500 | -0.023 | -0.019 | -0.020 | 0.117 | 0.113 | 0.112 | 0.116 | 0.112 | 0.110 |
| 10 | 200 | -0.012 | -0.010 | -0.011 | 0.114 | 0.112 | 0.111 | 0.113 | 0.110 | 0.108 |
|  | 500 | -0.014 | -0.015 | -0.015 | 0.076 | 0.073 | 0.072 | 0.075 | 0.073 | 0.071 |
| 20 | 200 | -0.010 | -0.007 | -0.010 | 0.074 | 0.071 | 0.071 | 0.073 | 0.071 | 0.071 |
|  | 500 | -0.005 | -0.005 | -0.005 | 0.072 | 0.071 | 0.071 | 0.072 | 0.071 | 0.070 |
|  |  | *Specificity* | | | | | | | | |
| 5 | 200 | -0.037 | -0.033 | -0.034 | 0.122 | 0.117 | 0.115 | 0.121 | 0.116 | 0.113 |
|  | 500 | -0.021 | -0.022 | -0.021 | 0.116 | 0.115 | 0.112 | 0.115 | 0.113 | 0.111 |
| 10 | 200 | -0.010 | -0.011 | -0.012 | 0.113 | 0.113 | 0.112 | 0.112 | 0.111 | 0.109 |
|  | 500 | -0.016 | -0.015 | -0.015 | 0.076 | 0.073 | 0.072 | 0.076 | 0.073 | 0.071 |
| 20 | 200 | -0.009 | -0.010 | -0.008 | 0.073 | 0.072 | 0.071 | 0.073 | 0.072 | 0.070 |
|  | 500 | -0.005 | -0.004 | -0.005 | 0.072 | 0.071 | 0.070 | 0.071 | 0.070 | 0.069 |
|  |  | *Classification Consistency* | | | | | | | | |
| 5 | 200 | -0.046 | -0.044 | -0.044 | 0.066 | 0.054 | 0.043 | 0.066 | 0.053 | 0.043 |
|  | 500 | -0.026 | -0.025 | -0.025 | 0.051 | 0.041 | 0.032 | 0.051 | 0.041 | 0.032 |
| 10 | 200 | -0.012 | -0.011 | -0.013 | 0.041 | 0.032 | 0.026 | 0.040 | 0.032 | 0.025 |
|  | 500 | -0.019 | -0.020 | -0.019 | 0.038 | 0.031 | 0.024 | 0.038 | 0.031 | 0.024 |
| 20 | 200 | -0.012 | -0.011 | -0.011 | 0.030 | 0.024 | 0.019 | 0.030 | 0.023 | 0.018 |
|  | 500 | -0.006 | -0.005 | -0.006 | 0.024 | 0.019 | 0.015 | 0.024 | 0.019 | 0.015 |

**Note***:* i is for number of items, n is for sample size, and f is for average factor loading

Table B2. *Bayesian credible interval coverage (diffused priors) and balance [lower, upper] for a cutpoint at .75SD*

|  |  | 95% Equal-tail bootstrap confidence interval | | | 95% HPD Bootstrap confidence interval | | |
| --- | --- | --- | --- | --- | --- | --- | --- |
| I | n | *f* = .5 | *f* = .6 | *f* = .7 | *f* = .5 | *f* = .6 | *f* = .7 |
|  |  | *Classification Rate* | | | | | |
| 5 | 200 | **0.236[0.000,0.764]** | **0.066[0.000,0.934]** | **0.002[0.000,0.998]** | **0.260[0.000,0.740]** | **0.080[0.000,0.920]** | **0.005[0.000,0.995]** |
|  | 500 | **0.391[0.000,0.609]** | **0.196[0.000,0.804]** | **0.046[0.000,0.954]** | **0.415[0.000,0.585]** | **0.243[0.000,0.757]** | **0.096[0.000,0.904]** |
| 10 | 200 | **0.653[0.000,0.347]** | **0.519[0.000,0.481]** | **0.183[0.000,0.817]** | **0.709[0.000,0.291]** | **0.629[0.000,0.371]** | **0.364[0.000,0.636]** |
|  | 500 | **0.578[0.000,0.422]** | **0.313[0.000,0.687]** | **0.072[0.000,0.928]** | **0.587[0.000,0.413]** | **0.326[0.000,0.674]** | **0.095[0.000,0.905]** |
| 20 | 200 | **0.654[0.000,0.346]** | **0.544[0.000,0.456]** | **0.338[0.000,0.662]** | **0.662[0.000,0.338]** | **0.557[0.000,0.443]** | **0.438[0.000,0.562]** |
|  | 500 | **0.828[0.000,0.172]** | **0.777[0.000,0.223]** | **0.469[0.000,0.531]** | **0.841[0.000,0.159]** | **0.818[0.000,0.182]** | **0.575[0.000,0.425]** |
|  |  | *Sensitivity* | | | | | |
| 5 | 200 | **0.895[0.002,0.103]** | **0.885[0.005,0.110]** | **0.882[0.001,0.117]** | **0.884[0.005,0.111]** | **0.885[0.007,0.108]** | **0.882[0.003,0.115]** |
|  | 500 | 0.945[0.010,0.045] | 0.938[0.006,0.056] | 0.946[0.010,0.044] | 0.941[0.012,0.047] | 0.938[0.011,0.051] | 0.946[0.012,0.042] |
| 10 | 200 | 0.952[0.028,0.020] | 0.965[0.025,0.010] | 0.954[0.032,0.014] | 0.939[0.040,0.021] | 0.954[0.039,0.007] | 0.926[0.063,0.011] |
|  | 500 | **0.912[0.009,0.079]** | 0.933[0.006,0.061] | **0.920[0.005,0.075]** | **0.913[0.010,0.077]** | 0.930[0.007,0.063] | **0.921[0.008,0.071]** |
| 20 | 200 | 0.945[0.010,0.045] | 0.950[0.013,0.037] | 0.948[0.017,0.035] | 0.942[0.012,0.046] | 0.946[0.019,0.035] | 0.949[0.021,0.030] |
|  | 500 | 0.961[0.023,0.016] | 0.958[0.027,0.015] | 0.954[0.030,0.016] | 0.955[0.029,0.016] | 0.955[0.031,0.014] | 0.939[0.045,0.016] |
|  |  | *Specificity* | | | | | |
| 5 | 200 | **0.865[0.000,0.135]** | **0.855[0.005,0.140]** | **0.836[0.002,0.162]** | **0.882[0.001,0.117]** | **0.879[0.006,0.115]** | **0.870[0.005,0.125]** |
|  | 500 | **0.904[0.005,0.091]** | **0.899[0.000,0.101]** | **0.902[0.007,0.091]** | **0.916[0.008,0.076]** | **0.915[0.003,0.082]** | **0.924[0.011,0.065]** |
| 10 | 200 | **0.915[0.003,0.082]** | **0.915[0.005,0.080]** | **0.884[0.002,0.114]** | 0.934[0.004,0.062] | 0.932[0.006,0.062] | **0.923[0.004,0.073]** |
|  | 500 | 0.926[0.004,0.070] | 0.929[0.003,0.068] | **0.911[0.004,0.085]** | 0.932[0.004,0.064] | 0.941[0.004,0.055] | 0.928[0.006,0.066] |
| 20 | 200 | 0.939[0.010,0.051] | **0.907[0.006,0.087]** | 0.931[0.011,0.058] | 0.944[0.012,0.044] | **0.922[0.010,0.068]** | 0.938[0.015,0.047] |
|  | 500 | 0.950[0.005,0.045] | 0.945[0.006,0.049] | **0.924[0.004,0.072]** | 0.955[0.006,0.039] | 0.958[0.009,0.033] | 0.941[0.008,0.051] |
|  |  | *Classification Consistency* | | | | | |
| 5 | 200 | **0.542[0.000,0.458]** | **0.358[0.000,0.642]** | **0.106[0.000,0.894]** | **0.551[0.000,0.449]** | **0.368[0.000,0.632]** | **0.110[0.000,0.890]** |
|  | 500 | **0.696[0.000,0.304]** | **0.502[0.000,0.498]** | **0.325[0.000,0.675]** | **0.700[0.000,0.300]** | **0.515[0.000,0.485]** | **0.321[0.000,0.679]** |
| 10 | 200 | **0.843[0.000,0.157]** | **0.711[0.000,0.289]** | **0.518[0.000,0.482]** | **0.841[0.000,0.159]** | **0.711[0.000,0.289]** | **0.518[0.000,0.482]** |
|  | 500 | **0.732[0.000,0.268]** | **0.597[0.000,0.403]** | **0.343[0.000,0.657]** | **0.733[0.000,0.267]** | **0.599[0.000,0.401]** | **0.353[0.000,0.647]** |
| 20 | 200 | **0.836[0.000,0.164]** | **0.740[0.000,0.260]** | **0.657[0.000,0.343]** | **0.828[0.000,0.172]** | **0.738[0.000,0.262]** | **0.649[0.000,0.351]** |
|  | 500 | **0.924[0.002,0.074]** | **0.849[0.001,0.150]** | **0.739[0.000,0.261]** | **0.920[0.002,0.078]** | **0.847[0.001,0.152]** | **0.735[0.000,0.265]** |

**Note***:* i is for number of items, n is for sample size, and f is for average factor loading, and in bold are intervals outside the robustness criterion

Table B3. *Relative bias and width of the Bayesian credible intervals (diffused priors) for cutpoint at .75SD*

|  |  | Relative bias | | | 95% equal-tail interval width | | | 95% HPD interval width | | |
| --- | --- | --- | --- | --- | --- | --- | --- | --- | --- | --- |
| i | n | *f* = .5 | *f* = .6 | *f* = .7 | *f* = .5 | *f* = .6 | *f* = .7 | *f* = .5 | *f* = .6 | *f* = .7 |
|  |  | *Classification Rate* | | | | | | | | |
| 5 | 200 | -0.027 | -0.026 | -0.027 | 0.037 | 0.030 | 0.027 | 0.036 | 0.030 | 0.026 |
|  | 500 | -0.018 | -0.017 | -0.017 | 0.029 | 0.026 | 0.026 | 0.029 | 0.026 | 0.025 |
| 10 | 200 | -0.011 | -0.011 | -0.013 | 0.027 | 0.027 | 0.030 | 0.026 | 0.026 | 0.028 |
|  | 500 | -0.011 | -0.011 | -0.012 | 0.020 | 0.017 | 0.014 | 0.020 | 0.017 | 0.014 |
| 20 | 200 | -0.008 | -0.007 | -0.007 | 0.016 | 0.014 | 0.013 | 0.016 | 0.014 | 0.013 |
|  | 500 | -0.005 | -0.005 | -0.006 | 0.014 | 0.013 | 0.014 | 0.014 | 0.013 | 0.013 |
|  |  | *Sensitivity* | | | | | | | | |
| 5 | 200 | -0.065 | -0.058 | -0.055 | 0.214 | 0.207 | 0.202 | 0.213 | 0.206 | 0.200 |
|  | 500 | -0.030 | -0.025 | -0.023 | 0.206 | 0.200 | 0.196 | 0.205 | 0.198 | 0.193 |
| 10 | 200 | 0.002 | 0.007 | 0.003 | 0.199 | 0.191 | 0.185 | 0.197 | 0.188 | 0.180 |
|  | 500 | -0.025 | -0.025 | -0.023 | 0.134 | 0.132 | 0.129 | 0.134 | 0.131 | 0.128 |
| 20 | 200 | -0.014 | -0.008 | -0.011 | 0.132 | 0.129 | 0.128 | 0.131 | 0.128 | 0.127 |
|  | 500 | 0.000 | 0.002 | 0.000 | 0.129 | 0.128 | 0.126 | 0.129 | 0.127 | 0.124 |
|  |  | *Specificity* | | | | | | | | |
| 5 | 200 | -0.017 | -0.016 | -0.017 | 0.068 | 0.067 | 0.066 | 0.067 | 0.066 | 0.065 |
|  | 500 | -0.013 | -0.014 | -0.013 | 0.067 | 0.067 | 0.068 | 0.066 | 0.066 | 0.066 |
| 10 | 200 | -0.013 | -0.014 | -0.015 | 0.070 | 0.071 | 0.072 | 0.068 | 0.069 | 0.070 |
|  | 500 | -0.008 | -0.007 | -0.008 | 0.042 | 0.041 | 0.041 | 0.042 | 0.041 | 0.041 |
| 20 | 200 | -0.005 | -0.007 | -0.005 | 0.042 | 0.042 | 0.041 | 0.041 | 0.041 | 0.040 |
|  | 500 | -0.005 | -0.005 | -0.006 | 0.042 | 0.042 | 0.043 | 0.042 | 0.041 | 0.042 |
|  |  | *Classification Consistency* | | | | | | | | |
| 5 | 200 | -0.031 | -0.030 | -0.031 | 0.054 | 0.044 | 0.036 | 0.053 | 0.044 | 0.036 |
|  | 500 | -0.019 | -0.019 | -0.018 | 0.042 | 0.034 | 0.027 | 0.041 | 0.034 | 0.027 |
| 10 | 200 | -0.011 | -0.011 | -0.012 | 0.033 | 0.026 | 0.021 | 0.033 | 0.026 | 0.021 |
|  | 500 | -0.014 | -0.014 | -0.014 | 0.031 | 0.025 | 0.020 | 0.031 | 0.025 | 0.020 |
| 20 | 200 | -0.008 | -0.008 | -0.007 | 0.025 | 0.020 | 0.016 | 0.025 | 0.020 | 0.016 |
|  | 500 | -0.005 | -0.005 | -0.005 | 0.020 | 0.016 | 0.013 | 0.020 | 0.016 | 0.013 |

**Note***:* i is for number of items, n is for sample size, and f is for average factor loading

Table B4. *Bayesian credible interval coverage (diffused priors) and balance [lower, upper] for a cutpoint at 1.5SD*

|  |  | 95% Equal-tail Bayesian credible interval | | | 95% HPD Bayesian credible interval | | |
| --- | --- | --- | --- | --- | --- | --- | --- |
| I | n | *f* = .5 | *f* = .6 | *f* = .7 | *f* = .5 | *f* = .6 | *f* = .7 |
|  |  | *Classification Rate* | | | | | |
| 5 | 200 | **0.190[0.000,0.810]** | **0.023[0.000,0.977]** | **0.000[0.000,1.000]** | **0.278[0.000,0.722]** | **0.058[0.000,0.942]** | **0.002[0.000,0.998]** |
|  | 500 | **0.186[0.000,0.814]** | **0.080[0.000,0.920]** | **0.001[0.000,0.999]** | **0.322[0.000,0.678]** | **0.208[0.000,0.792]** | **0.010[0.000,0.990]** |
| 10 | 200 | **0.390[0.000,0.610]** | **0.335[0.000,0.665]** | **0.009[0.000,0.991]** | **0.614[0.000,0.386]** | **0.630[0.000,0.370]** | **0.098[0.000,0.902]** |
|  | 500 | **0.631[0.000,0.369]** | **0.312[0.000,0.688]** | **0.084[0.000,0.916]** | **0.706[0.000,0.294]** | **0.418[0.000,0.582]** | **0.162[0.000,0.838]** |
| 20 | 200 | **0.536[0.000,0.464]** | **0.521[0.000,0.479]** | **0.085[0.000,0.915]** | **0.671[0.000,0.329]** | **0.669[0.000,0.331]** | **0.217[0.000,0.783]** |
|  | 500 | **0.755[0.000,0.245]** | **0.822[0.000,0.178]** | **0.171[0.000,0.829]** | **0.875[0.000,0.125]** | 0.936[0.000,0.064] | **0.409[0.000,0.591]** |
|  |  | *Sensitivity* | | | | | |
| 5 | 200 | **0.908[0.004,0.088]** | **0.920[0.005,0.075]** | **0.910[0.002,0.088]** | **0.892[0.006,0.102]** | **0.908[0.005,0.087]** | **0.907[0.004,0.089]** |
|  | 500 | 0.956[0.012,0.032] | 0.944[0.011,0.045] | 0.945[0.019,0.036] | 0.944[0.016,0.040] | 0.930[0.022,0.048] | 0.936[0.027,0.037] |
| 10 | 200 | 0.948[0.046,0.006] | 0.949[0.043,0.008] | 0.929[0.061,0.010] | **0.918[0.073,0.009]** | **0.908[0.084,0.008]** | **0.876[0.114,0.010]** |
|  | 500 | 0.929[0.011,0.060] | 0.944[0.009,0.047] | 0.931[0.009,0.060] | 0.929[0.010,0.061] | 0.934[0.014,0.052] | 0.928[0.012,0.060] |
| 20 | 200 | 0.955[0.011,0.034] | 0.960[0.016,0.024] | 0.950[0.021,0.029] | 0.949[0.013,0.038] | 0.952[0.024,0.024] | 0.946[0.025,0.029] |
|  | 500 | 0.952[0.037,0.011] | 0.945[0.043,0.012] | 0.936[0.054,0.010] | 0.939[0.047,0.014] | 0.937[0.051,0.012] | **0.917[0.072,0.011]** |
|  |  | *Specificity* | | | | | |
| 5 | 200 | **0.917[0.001,0.082]** | **0.891[0.006,0.103]** | **0.902[0.003,0.095]** | 0.942[0.004,0.054] | 0.926[0.010,0.064] | 0.937[0.006,0.057] |
|  | 500 | **0.902[0.005,0.093]** | **0.907[0.002,0.091]** | **0.905[0.003,0.092]** | 0.931[0.005,0.064] | 0.942[0.004,0.054] | 0.939[0.010,0.051] |
| 10 | 200 | **0.863[0.001,0.136]** | **0.877[0.002,0.121]** | **0.837[0.002,0.161]** | **0.917[0.003,0.080]** | 0.927[0.006,0.067] | **0.907[0.005,0.088]** |
|  | 500 | 0.950[0.004,0.046] | 0.946[0.002,0.052] | 0.931[0.007,0.062] | 0.951[0.011,0.038] | 0.956[0.007,0.037] | 0.944[0.014,0.042] |
| 20 | 200 | 0.940[0.007,0.053] | **0.921[0.005,0.074]** | 0.948[0.006,0.046] | 0.952[0.011,0.037] | 0.948[0.008,0.044] | 0.960[0.012,0.028] |
|  | 500 | **0.910[0.001,0.089]** | 0.937[0.004,0.059] | **0.908[0.002,0.090]** | 0.944[0.002,0.054] | 0.954[0.006,0.040] | 0.940[0.004,0.056] |
|  |  | *Classification Consistency* | | | | | |
| 5 | 200 | **0.863[0.000,0.137]** | **0.776[0.001,0.223]** | **0.609[0.000,0.391]** | **0.864[0.001,0.135]** | **0.778[0.002,0.220]** | **0.625[0.000,0.375]** |
|  | 500 | **0.861[0.003,0.136]** | **0.787[0.000,0.213]** | **0.685[0.000,0.315]** | **0.862[0.003,0.135]** | **0.794[0.000,0.206]** | **0.692[0.001,0.307]** |
| 10 | 200 | **0.797[0.000,0.203]** | **0.770[0.000,0.230]** | **0.621[0.000,0.379]** | **0.795[0.000,0.205]** | **0.776[0.000,0.224]** | **0.634[0.001,0.365]** |
|  | 500 | **0.921[0.002,0.077]** | **0.851[0.000,0.149]** | **0.769[0.000,0.231]** | **0.920[0.002,0.078]** | **0.853[0.000,0.147]** | **0.773[0.000,0.227]** |
| 20 | 200 | 0.930[0.004,0.066] | **0.857[0.003,0.140]** | **0.841[0.001,0.158]** | 0.926[0.007,0.067] | **0.857[0.003,0.140]** | **0.844[0.001,0.155]** |
|  | 500 | **0.889[0.001,0.110]** | **0.897[0.001,0.102]** | **0.812[0.000,0.188]** | **0.893[0.001,0.106]** | **0.903[0.002,0.095]** | **0.817[0.000,0.183]** |

**Note***:* i is for number of items, n is for sample size, and f is for average factor loading, and in bold are intervals outside the robustness criterion

Table B5. *Relative bias and width of the Bayesian credible intervals (diffused priors) with cutpoint at 1.5SD*

|  |  | Relative bias | | | 95% equal-tail interval width | | | 95% HPD interval width | | |
| --- | --- | --- | --- | --- | --- | --- | --- | --- | --- | --- |
| i | n | *f* = .5 | *f* = .6 | *f* = .7 | *f* = .5 | *f* = .6 | *f* = .7 | *f* = .5 | *f* = .6 | *f* = .7 |
|  |  | *Classification Rate* | | | | | | | | |
| 5 | 200 | -0.010 | -0.011 | -0.011 | 0.018 | 0.017 | 0.016 | 0.018 | 0.016 | 0.015 |
|  | 500 | -0.008 | -0.008 | -0.009 | 0.017 | 0.017 | 0.019 | 0.016 | 0.016 | 0.017 |
| 10 | 200 | -0.007 | -0.007 | -0.009 | 0.020 | 0.022 | 0.025 | 0.019 | 0.020 | 0.022 |
|  | 500 | -0.004 | -0.005 | -0.005 | 0.010 | 0.009 | 0.008 | 0.010 | 0.008 | 0.008 |
| 20 | 200 | -0.004 | -0.003 | -0.004 | 0.009 | 0.008 | 0.008 | 0.008 | 0.008 | 0.008 |
|  | 500 | -0.003 | -0.002 | -0.004 | 0.009 | 0.009 | 0.010 | 0.008 | 0.008 | 0.009 |
|  |  | *Sensitivity* | | | | | | | | |
| 5 | 200 | -0.099 | -0.083 | -0.078 | 0.316 | 0.320 | 0.322 | 0.314 | 0.318 | 0.320 |
|  | 500 | -0.034 | -0.028 | -0.023 | 0.321 | 0.321 | 0.318 | 0.319 | 0.318 | 0.312 |
| 10 | 200 | 0.033 | 0.038 | 0.030 | 0.316 | 0.306 | 0.292 | 0.313 | 0.300 | 0.281 |
|  | 500 | -0.038 | -0.036 | -0.031 | 0.206 | 0.210 | 0.213 | 0.205 | 0.209 | 0.211 |
| 20 | 200 | -0.017 | -0.007 | -0.010 | 0.210 | 0.212 | 0.215 | 0.209 | 0.211 | 0.213 |
|  | 500 | 0.013 | 0.015 | 0.012 | 0.212 | 0.212 | 0.211 | 0.211 | 0.211 | 0.208 |
|  |  | *Specificity* | | | | | | | | |
| 5 | 200 | -0.006 | -0.007 | -0.007 | 0.036 | 0.036 | 0.035 | 0.035 | 0.035 | 0.034 |
|  | 500 | -0.007 | -0.007 | -0.007 | 0.037 | 0.037 | 0.037 | 0.036 | 0.035 | 0.035 |
| 10 | 200 | -0.009 | -0.009 | -0.01 | 0.041 | 0.042 | 0.043 | 0.039 | 0.04 | 0.04 |
|  | 500 | -0.003 | -0.003 | -0.003 | 0.022 | 0.021 | 0.021 | 0.022 | 0.021 | 0.021 |
| 20 | 200 | -0.003 | -0.004 | -0.003 | 0.022 | 0.022 | 0.021 | 0.021 | 0.021 | 0.021 |
|  | 500 | -0.004 | -0.003 | -0.004 | 0.023 | 0.023 | 0.023 | 0.022 | 0.022 | 0.022 |
|  |  | *Classification Consistency* | | | | | | | | |
| 5 | 200 | -0.011 | -0.013 | -0.013 | 0.045 | 0.037 | 0.030 | 0.045 | 0.037 | 0.030 |
|  | 500 | -0.009 | -0.010 | -0.010 | 0.037 | 0.030 | 0.024 | 0.037 | 0.030 | 0.024 |
| 10 | 200 | -0.009 | -0.008 | -0.008 | 0.030 | 0.024 | 0.019 | 0.030 | 0.024 | 0.019 |
|  | 500 | -0.005 | -0.006 | -0.006 | 0.027 | 0.022 | 0.017 | 0.026 | 0.022 | 0.017 |
| 20 | 200 | -0.003 | -0.005 | -0.004 | 0.022 | 0.018 | 0.014 | 0.022 | 0.018 | 0.014 |
|  | 500 | -0.004 | -0.003 | -0.004 | 0.018 | 0.014 | 0.011 | 0.018 | 0.014 | 0.011 |

**Note***:* i is for number of items, n is for sample size, and f is for average factor loading

Table C1. *Relative bias and width of the Bayesian credible intervals (informative priors) with cutpoint at mean*

|  |  | Relative bias | | | 95% equal-tail interval width | | | 95% HPD interval width | | |
| --- | --- | --- | --- | --- | --- | --- | --- | --- | --- | --- |
| i | n | *f* = .5 | *f* = .6 | *f* = .7 | *f* = .5 | *f* = .6 | *f* = .7 | *f* = .5 | *f* = .6 | *f* = .7 |
|  |  | *Classification Rate* | | | | | | | | |
| 5 | 200 | -0.003 | -0.004 | -0.004 | 0.045 | 0.037 | 0.032 | 0.045 | 0.036 | 0.031 |
|  | 500 | -0.002 | -0.003 | -0.004 | 0.036 | 0.031 | 0.030 | 0.036 | 0.031 | 0.029 |
| 10 | 200 | 0.002 | 0.000 | -0.002 | 0.032 | 0.030 | 0.031 | 0.031 | 0.029 | 0.029 |
|  | 500 | -0.001 | -0.002 | -0.002 | 0.028 | 0.022 | 0.018 | 0.028 | 0.022 | 0.017 |
| 20 | 200 | -0.001 | -0.001 | -0.002 | 0.022 | 0.018 | 0.016 | 0.022 | 0.018 | 0.016 |
|  | 500 | 0.000 | 0.000 | -0.001 | 0.018 | 0.016 | 0.015 | 0.018 | 0.016 | 0.015 |
|  |  | *Sensitivity* | | | | | | | | |
| 5 | 200 | -0.002 | -0.003 | -0.002 | 0.118 | 0.114 | 0.112 | 0.117 | 0.113 | 0.110 |
|  | 500 | -0.001 | 0.000 | -0.002 | 0.114 | 0.111 | 0.110 | 0.113 | 0.109 | 0.108 |
| 10 | 200 | 0.002 | 0.002 | 0.000 | 0.112 | 0.109 | 0.108 | 0.110 | 0.107 | 0.104 |
|  | 500 | 0.000 | -0.001 | -0.001 | 0.075 | 0.073 | 0.071 | 0.074 | 0.072 | 0.071 |
| 20 | 200 | -0.001 | 0.001 | -0.002 | 0.073 | 0.071 | 0.071 | 0.072 | 0.070 | 0.070 |
|  | 500 | 0.001 | 0.000 | 0.000 | 0.071 | 0.070 | 0.070 | 0.071 | 0.070 | 0.069 |
|  |  | *Specificity* | | | | | | | | |
| 5 | 200 | -0.002 | -0.001 | -0.003 | 0.117 | 0.114 | 0.112 | 0.116 | 0.112 | 0.110 |
|  | 500 | 0.000 | -0.003 | -0.002 | 0.113 | 0.113 | 0.110 | 0.112 | 0.111 | 0.108 |
| 10 | 200 | 0.004 | 0.001 | -0.001 | 0.111 | 0.110 | 0.109 | 0.109 | 0.108 | 0.106 |
|  | 500 | -0.002 | -0.002 | -0.001 | 0.075 | 0.073 | 0.071 | 0.075 | 0.072 | 0.071 |
| 20 | 200 | 0.000 | -0.002 | 0.000 | 0.073 | 0.072 | 0.070 | 0.072 | 0.071 | 0.069 |
|  | 500 | 0.001 | 0.001 | 0.000 | 0.071 | 0.070 | 0.070 | 0.071 | 0.069 | 0.069 |
|  |  | *Classification Consistency* | | | | | | | | |
| 5 | 200 | 0.002 | 0.001 | 0.001 | 0.056 | 0.044 | 0.034 | 0.056 | 0.044 | 0.033 |
|  | 500 | 0.004 | 0.003 | 0.003 | 0.045 | 0.035 | 0.027 | 0.044 | 0.035 | 0.027 |
| 10 | 200 | 0.008 | 0.007 | 0.004 | 0.036 | 0.028 | 0.022 | 0.036 | 0.028 | 0.021 |
|  | 500 | 0.001 | 0.000 | 0.000 | 0.036 | 0.028 | 0.021 | 0.035 | 0.028 | 0.021 |
| 20 | 200 | 0.001 | 0.001 | 0.001 | 0.028 | 0.022 | 0.017 | 0.028 | 0.022 | 0.017 |
|  | 500 | 0.003 | 0.003 | 0.001 | 0.023 | 0.018 | 0.014 | 0.022 | 0.017 | 0.013 |

**Note***:* i is for number of items, n is for sample size, and f is for average factor loading

Table C2. *Bayesian credible interval coverage (informative priors) and balance [lower, upper] for a cutpoint at .75SD*

|  |  | 95% Equal-tail bootstrap confidence interval | | | 95% HPD Bootstrap confidence interval | | |
| --- | --- | --- | --- | --- | --- | --- | --- |
| I | n | *f* = .5 | *f* = .6 | *f* = .7 | *f* = .5 | *f* = .6 | *f* = .7 |
|  |  | *Classification Rate* | | | | | |
| 5 | 200 | 0.961[0.005,0.034] | 0.957[0.000,0.043] | 0.947[0.000,0.053] | 0.961[0.012,0.027] | 0.963[0.000,0.037] | 0.966[0.000,0.034] |
|  | 500 | 0.965[0.003,0.032] | 0.960[0.000,0.040] | 0.969[0.000,0.031] | 0.968[0.005,0.027] | 0.975[0.000,0.025] | **0.989[0.000,0.011]** |
| 10 | 200 | **0.986[0.001,0.013]** | **0.982[0.000,0.018]** | 0.965[0.000,0.035] | **0.990[0.003,0.007]** | **0.996[0.000,0.004]** | **0.986[0.000,0.014]** |
|  | 500 | 0.967[0.012,0.021] | 0.964[0.006,0.030] | 0.949[0.000,0.051] | 0.969[0.013,0.018] | 0.960[0.012,0.028] | 0.957[0.001,0.042] |
| 20 | 200 | 0.975[0.005,0.020] | 0.972[0.000,0.028] | 0.974[0.000,0.026] | **0.979[0.005,0.016]** | **0.978[0.001,0.021]** | **0.990[0.000,0.010]** |
|  | 500 | **0.988[0.003,0.009]** | **0.983[0.001,0.016]** | 0.970[0.000,0.030] | **0.987[0.006,0.007]** | **0.988[0.002,0.010]** | **0.988[0.000,0.012]** |
|  |  | *Sensitivity* | | | | | |
| 5 | 200 | 0.957[0.025,0.018] | 0.940[0.033,0.027] | 0.949[0.027,0.024] | 0.952[0.031,0.017] | 0.931[0.041,0.028] | 0.937[0.045,0.018] |
|  | 500 | 0.946[0.037,0.017] | 0.943[0.043,0.014] | 0.945[0.037,0.018] | 0.937[0.047,0.016] | **0.924[0.062,0.014]** | **0.923[0.059,0.018]** |
| 10 | 200 | **0.921[0.071,0.008]** | 0.926[0.067,0.007] | **0.914[0.078,0.008]** | **0.902[0.090,0.008]** | **0.890[0.104,0.006]** | **0.867[0.126,0.007]** |
|  | 500 | 0.945[0.029,0.026] | 0.955[0.022,0.023] | 0.951[0.026,0.023] | 0.939[0.033,0.028] | 0.952[0.027,0.021] | 0.939[0.037,0.024] |
| 20 | 200 | 0.956[0.022,0.022] | 0.948[0.036,0.016] | 0.951[0.031,0.018] | 0.952[0.026,0.022] | 0.936[0.048,0.016] | 0.936[0.045,0.019] |
|  | 500 | 0.943[0.046,0.011] | 0.949[0.042,0.009] | 0.928[0.063,0.009] | 0.935[0.058,0.007] | 0.935[0.056,0.009] | **0.900[0.091,0.009]** |
|  |  | *Specificity* | | | | | |
| 5 | 200 | 0.963[0.012,0.025] | 0.944[0.020,0.036] | 0.948[0.020,0.032] | 0.958[0.024,0.018] | 0.940[0.034,0.026] | 0.948[0.034,0.018] |
|  | 500 | 0.952[0.018,0.030] | 0.948[0.017,0.035] | 0.951[0.018,0.031] | 0.948[0.027,0.025] | 0.952[0.025,0.023] | 0.956[0.025,0.019] |
| 10 | 200 | 0.936[0.020,0.044] | 0.952[0.011,0.037] | 0.937[0.006,0.057] | 0.937[0.030,0.033] | 0.959[0.017,0.024] | 0.951[0.016,0.033] |
|  | 500 | 0.959[0.018,0.023] | 0.956[0.020,0.024] | 0.950[0.023,0.027] | 0.954[0.026,0.020] | 0.952[0.031,0.017] | 0.949[0.034,0.017] |
| 20 | 200 | 0.952[0.029,0.019] | 0.951[0.014,0.035] | 0.956[0.019,0.025] | 0.951[0.034,0.015] | 0.945[0.024,0.031] | 0.953[0.029,0.018] |
|  | 500 | 0.967[0.010,0.023] | 0.962[0.013,0.025] | 0.945[0.010,0.045] | 0.970[0.015,0.015] | 0.960[0.021,0.019] | 0.949[0.021,0.030] |
|  |  | *Classification Consistency* | | | | | |
| 5 | 200 | 0.951[0.027,0.022] | 0.952[0.038,0.010] | 0.951[0.034,0.015] | 0.952[0.028,0.020] | 0.950[0.039,0.011] | 0.944[0.041,0.015] |
|  | 500 | 0.952[0.036,0.012] | 0.964[0.024,0.012] | 0.959[0.033,0.008] | 0.947[0.041,0.012] | 0.964[0.022,0.014] | 0.957[0.034,0.009] |
| 10 | 200 | 0.943[0.054,0.003] | 0.945[0.048,0.007] | 0.963[0.027,0.010] | 0.939[0.057,0.004] | 0.942[0.050,0.008] | 0.958[0.031,0.011] |
|  | 500 | 0.958[0.021,0.021] | 0.959[0.021,0.020] | 0.965[0.023,0.012] | 0.959[0.022,0.019] | 0.959[0.025,0.016] | 0.960[0.028,0.012] |
| 20 | 200 | 0.966[0.022,0.012] | 0.957[0.025,0.018] | 0.957[0.037,0.006] | 0.961[0.027,0.012] | 0.961[0.025,0.014] | 0.953[0.039,0.008] |
|  | 500 | 0.960[0.031,0.009] | 0.954[0.033,0.013] | 0.965[0.022,0.013] | 0.957[0.035,0.008] | 0.950[0.036,0.014] | 0.965[0.022,0.013] |

**Note***:* i is for number of items, n is for sample size, and f is for average factor loading, and in bold are intervals outside the robustness criterion

Table C3. *Relative bias and width of the Bayesian credible intervals (informative priors) for cutpoint at .75SD*

|  |  | Relative bias | | | 95% equal-tail interval width | | | 95% HPD interval width | | |
| --- | --- | --- | --- | --- | --- | --- | --- | --- | --- | --- |
| i | n | *f* = .5 | *f* = .6 | *f* = .7 | *f* = .5 | *f* = .6 | *f* = .7 | *f* = .5 | *f* = .6 | *f* = .7 |
|  |  | *Classification Rate* | | | | | | | | |
| 5 | 200 | -0.002 | -0.003 | -0.004 | 0.031 | 0.027 | 0.026 | 0.031 | 0.027 | 0.025 |
|  | 500 | -0.002 | -0.003 | -0.003 | 0.027 | 0.025 | 0.026 | 0.026 | 0.025 | 0.025 |
| 10 | 200 | -0.001 | -0.002 | -0.004 | 0.026 | 0.027 | 0.031 | 0.025 | 0.025 | 0.028 |
|  | 500 | -0.001 | -0.002 | -0.002 | 0.019 | 0.016 | 0.013 | 0.019 | 0.015 | 0.013 |
| 20 | 200 | -0.001 | -0.001 | -0.001 | 0.015 | 0.013 | 0.013 | 0.015 | 0.013 | 0.012 |
|  | 500 | 0.000 | -0.001 | -0.002 | 0.014 | 0.013 | 0.014 | 0.013 | 0.012 | 0.013 |
|  |  | *Sensitivity* | | | | | | | | |
| 5 | 200 | 0.000 | 0.000 | 0.001 | 0.208 | 0.202 | 0.198 | 0.206 | 0.201 | 0.195 |
|  | 500 | 0.008 | 0.009 | 0.008 | 0.202 | 0.196 | 0.192 | 0.200 | 0.194 | 0.188 |
| 10 | 200 | 0.026 | 0.027 | 0.021 | 0.194 | 0.185 | 0.178 | 0.191 | 0.182 | 0.172 |
|  | 500 | 0.002 | 0.000 | 0.001 | 0.133 | 0.130 | 0.128 | 0.132 | 0.130 | 0.127 |
| 20 | 200 | 0.002 | 0.006 | 0.003 | 0.130 | 0.127 | 0.127 | 0.130 | 0.126 | 0.126 |
|  | 500 | 0.010 | 0.010 | 0.008 | 0.127 | 0.125 | 0.123 | 0.127 | 0.124 | 0.121 |
|  |  | *Specificity* | | | | | | | | |
| 5 | 200 | -0.002 | -0.002 | -0.003 | 0.066 | 0.065 | 0.065 | 0.065 | 0.064 | 0.063 |
|  | 500 | -0.003 | -0.005 | -0.004 | 0.066 | 0.066 | 0.066 | 0.065 | 0.065 | 0.064 |
| 10 | 200 | -0.006 | -0.007 | -0.009 | 0.068 | 0.069 | 0.070 | 0.066 | 0.067 | 0.067 |
|  | 500 | -0.001 | -0.001 | -0.001 | 0.042 | 0.041 | 0.041 | 0.041 | 0.041 | 0.040 |
| 20 | 200 | 0.000 | -0.003 | -0.001 | 0.041 | 0.041 | 0.041 | 0.041 | 0.041 | 0.040 |
|  | 500 | -0.002 | -0.002 | -0.003 | 0.041 | 0.041 | 0.041 | 0.041 | 0.041 | 0.041 |
|  |  | *Classification Consistency* | | | | | | | | |
| 5 | 200 | 0.001 | 0.001 | 0.001 | 0.046 | 0.037 | 0.028 | 0.046 | 0.036 | 0.028 |
|  | 500 | 0.002 | 0.001 | 0.002 | 0.037 | 0.029 | 0.022 | 0.037 | 0.029 | 0.022 |
| 10 | 200 | 0.004 | 0.002 | 0.001 | 0.029 | 0.023 | 0.018 | 0.029 | 0.023 | 0.018 |
|  | 500 | 0.000 | 0.000 | 0.001 | 0.029 | 0.023 | 0.018 | 0.029 | 0.023 | 0.018 |
| 20 | 200 | 0.001 | 0.000 | 0.001 | 0.023 | 0.019 | 0.014 | 0.023 | 0.018 | 0.014 |
|  | 500 | 0.001 | 0.001 | 0.000 | 0.019 | 0.015 | 0.012 | 0.019 | 0.015 | 0.012 |

**Note***:* i is for number of items, n is for sample size, and f is for average factor loading

Table C4. *Bayesian credible interval coverage (informative priors) and balance [lower, upper] for a cutpoint at 1.5SD*

|  |  | 95% Equal-tail Bayesian credible interval | | | 95% HPD Bayesian credible interval | | |
| --- | --- | --- | --- | --- | --- | --- | --- |
| I | n | *f* = .5 | *f* = .6 | *f* = .7 | *f* = .5 | *f* = .6 | *f* = .7 |
|  |  | *Classification Rate* | | | | | |
| 5 | 200 | 0.963[0.006,0.031] | 0.953[0.000,0.047] | 0.964[0.000,0.036] | 0.974[0.006,0.020] | **0.983[0.000,0.017]** | **0.991[0.000,0.009]** |
|  | 500 | 0.941[0.000,0.059] | 0.957[0.000,0.043] | **0.918[0.000,0.082]** | **0.976[0.000,0.024]** | **0.986[0.000,0.014]** | **0.982[0.000,0.018]** |
| 10 | 200 | 0.962[0.000,0.038] | 0.970[0.000,0.030] | 0.869[0.000,0.131] | **0.984[0.000,0.016]** | **0.987[0.000,0.013]** | 0.971[0.000,0.029] |
|  | 500 | **0.976[0.006,0.018]** | 0.972[0.001,0.027] | **0.986[0.000,0.014]** | **0.980[0.009,0.011]** | **0.988[0.001,0.011]** | **0.995[0.000,0.005]** |
| 20 | 200 | 0.965[0.000,0.035] | **0.978[0.000,0.022]** | 0.933[0.000,0.067] | **0.980[0.001,0.019]** | **0.993[0.000,0.007]** | **0.978[0.000,0.022]** |
|  | 500 | **0.983[0.000,0.017]** | **0.990[0.000,0.010]** | 0.917[0.000,0.083] | **0.994[0.000,0.006]** | **0.997[0.000,0.003]** | **0.979[0.000,0.021]** |
|  |  | *Sensitivity* | | | | | |
| 5 | 200 | 0.949[0.029,0.022] | 0.942[0.035,0.023] | 0.951[0.029,0.020] | 0.940[0.035,0.025] | 0.927[0.045,0.028] | 0.929[0.050,0.021] |
|  | 500 | 0.943[0.044,0.013] | 0.944[0.045,0.011] | 0.939[0.046,0.015] | **0.919[0.063,0.018]** | **0.920[0.064,0.016]** | **0.900[0.086,0.014]** |
| 10 | 200 | **0.900[0.096,0.004]** | **0.900[0.096,0.004]** | **0.881[0.110,0.009]** | **0.872[0.123,0.005]** | **0.855[0.140,0.005]** | **0.813[0.179,0.008]** |
|  | 500 | 0.946[0.028,0.026] | 0.955[0.024,0.021] | 0.946[0.032,0.022] | 0.939[0.033,0.028] | 0.945[0.030,0.025] | 0.934[0.042,0.024] |
| 20 | 200 | 0.951[0.029,0.020] | 0.947[0.035,0.018] | 0.951[0.036,0.013] | 0.943[0.035,0.022] | 0.925[0.057,0.018] | 0.932[0.052,0.016] |
|  | 500 | 0.925[0.066,0.009] | 0.934[0.055,0.011] | **0.921[0.071,0.008]** | **0.921[0.072,0.007]** | **0.909[0.080,0.011]** | **0.894[0.098,0.008]** |
|  |  | *Specificity* | | | | | |
| 5 | 200 | 0.961[0.013,0.026] | 0.946[0.017,0.037] | 0.957[0.019,0.024] | 0.963[0.022,0.015] | 0.946[0.029,0.025] | 0.950[0.039,0.011] |
|  | 500 | 0.949[0.010,0.041] | 0.946[0.009,0.045] | 0.948[0.016,0.036] | 0.952[0.021,0.027] | 0.950[0.026,0.024] | 0.952[0.028,0.020] |
| 10 | 200 | **0.919[0.005,0.076]** | 0.928[0.006,0.066] | **0.898[0.006,0.096]** | 0.947[0.009,0.044] | 0.957[0.007,0.036] | 0.935[0.013,0.052] |
|  | 500 | 0.954[0.023,0.023] | 0.960[0.016,0.024] | 0.956[0.020,0.024] | 0.958[0.027,0.015] | 0.963[0.021,0.016] | 0.952[0.034,0.014] |
| 20 | 200 | 0.960[0.016,0.024] | 0.953[0.009,0.038] | 0.958[0.014,0.028] | 0.964[0.022,0.014] | 0.958[0.015,0.027] | 0.953[0.026,0.021] |
|  | 500 | 0.948[0.003,0.049] | 0.952[0.007,0.041] | 0.933[0.005,0.062] | 0.957[0.009,0.034] | 0.965[0.012,0.023] | 0.952[0.013,0.035] |
|  |  | *Classification Consistency* | | | | | |
| 5 | 200 | 0.961[0.015,0.024] | 0.945[0.017,0.038] | 0.954[0.020,0.026] | 0.959[0.018,0.023] | 0.946[0.019,0.035] | 0.951[0.025,0.024] |
|  | 500 | 0.954[0.015,0.031] | 0.949[0.015,0.036] | 0.952[0.015,0.033] | 0.952[0.019,0.029] | 0.947[0.017,0.036] | 0.954[0.016,0.030] |
| 10 | 200 | 0.940[0.010,0.050] | 0.956[0.007,0.037] | **0.918[0.007,0.075]** | 0.935[0.014,0.051] | 0.957[0.009,0.034] | **0.921[0.007,0.072]** |
|  | 500 | 0.959[0.022,0.019] | 0.964[0.009,0.027] | 0.961[0.015,0.024] | 0.954[0.026,0.020] | 0.963[0.012,0.025] | 0.961[0.015,0.024] |
| 20 | 200 | 0.963[0.023,0.014] | 0.953[0.013,0.034] | 0.960[0.012,0.028] | 0.955[0.028,0.017] | 0.951[0.014,0.035] | 0.959[0.014,0.027] |
|  | 500 | 0.965[0.008,0.027] | 0.968[0.008,0.024] | 0.940[0.004,0.056] | 0.967[0.008,0.025] | 0.965[0.009,0.026] | 0.939[0.006,0.055] |

**Note***:* i is for number of items, n is for sample size, and f is for average factor loading, and in bold are intervals outside the robustness criterion

Table C5. *Relative bias and width of the Bayesian credible intervals (informative priors) with cutpoint at 1.5SD*

|  |  | Relative bias | | | 95% equal-tail interval width | | | 95% HPD interval width | | |
| --- | --- | --- | --- | --- | --- | --- | --- | --- | --- | --- |
| i | n | *f* = .5 | *f* = .6 | *f* = .7 | *f* = .5 | *f* = .6 | *f* = .7 | *f* = .5 | *f* = .6 | *f* = .7 |
|  |  | *Classification Rate* | | | | | | | | |
| 5 | 200 | -0.002 | -0.002 | -0.002 | 0.017 | 0.016 | 0.017 | 0.016 | 0.015 | 0.015 |
|  | 500 | -0.003 | -0.002 | -0.003 | 0.016 | 0.017 | 0.019 | 0.015 | 0.016 | 0.017 |
| 10 | 200 | -0.003 | -0.003 | -0.005 | 0.020 | 0.022 | 0.025 | 0.018 | 0.019 | 0.022 |
|  | 500 | -0.001 | -0.001 | -0.001 | 0.009 | 0.008 | 0.008 | 0.009 | 0.008 | 0.008 |
| 20 | 200 | -0.001 | -0.001 | -0.002 | 0.008 | 0.008 | 0.008 | 0.008 | 0.008 | 0.008 |
|  | 500 | -0.001 | -0.001 | -0.002 | 0.009 | 0.009 | 0.010 | 0.008 | 0.008 | 0.009 |
|  |  | *Sensitivity* | | | | | | | | |
| 5 | 200 | 0.006 | 0.008 | 0.008 | 0.318 | 0.321 | 0.322 | 0.316 | 0.318 | 0.318 |
|  | 500 | 0.027 | 0.025 | 0.024 | 0.319 | 0.318 | 0.313 | 0.317 | 0.314 | 0.304 |
| 10 | 200 | 0.069 | 0.067 | 0.055 | 0.310 | 0.298 | 0.283 | 0.305 | 0.289 | 0.268 |
|  | 500 | 0.006 | 0.003 | 0.006 | 0.206 | 0.211 | 0.214 | 0.205 | 0.210 | 0.212 |
| 20 | 200 | 0.008 | 0.015 | 0.010 | 0.209 | 0.211 | 0.214 | 0.208 | 0.209 | 0.212 |
|  | 500 | 0.029 | 0.028 | 0.023 | 0.210 | 0.210 | 0.207 | 0.208 | 0.207 | 0.203 |
|  |  | *Specificity* | | | | | | | | |
| 5 | 200 | -0.002 | -0.002 | -0.002 | 0.035 | 0.035 | 0.035 | 0.034 | 0.033 | 0.033 |
|  | 500 | -0.003 | -0.004 | -0.004 | 0.036 | 0.036 | 0.036 | 0.035 | 0.034 | 0.034 |
| 10 | 200 | -0.007 | -0.007 | -0.008 | 0.039 | 0.040 | 0.041 | 0.038 | 0.038 | 0.038 |
|  | 500 | -0.001 | -0.001 | -0.001 | 0.022 | 0.021 | 0.021 | 0.021 | 0.021 | 0.020 |
| 20 | 200 | -0.001 | -0.002 | -0.001 | 0.021 | 0.021 | 0.021 | 0.021 | 0.021 | 0.020 |
|  | 500 | -0.003 | -0.002 | -0.003 | 0.022 | 0.022 | 0.022 | 0.021 | 0.021 | 0.021 |
|  |  | *Classification Consistency* | | | | | | | | |
| 5 | 200 | 0.000 | -0.001 | -0.001 | 0.039 | 0.031 | 0.024 | 0.038 | 0.031 | 0.024 |
|  | 500 | -0.001 | -0.002 | -0.001 | 0.032 | 0.026 | 0.020 | 0.032 | 0.026 | 0.020 |
| 10 | 200 | -0.003 | -0.002 | -0.002 | 0.027 | 0.021 | 0.016 | 0.027 | 0.021 | 0.016 |
|  | 500 | 0.000 | -0.001 | -0.001 | 0.025 | 0.020 | 0.015 | 0.025 | 0.020 | 0.015 |
| 20 | 200 | 0.000 | -0.001 | -0.001 | 0.021 | 0.017 | 0.013 | 0.021 | 0.016 | 0.013 |
|  | 500 | -0.001 | -0.001 | -0.001 | 0.017 | 0.014 | 0.011 | 0.017 | 0.013 | 0.010 |

**Note***:* i is for number of items, n is for sample size, and f is for average factor loading

Supplementary material

*Summary intervals for model-based classification accuracy and consistency indices*

# R code to estimate summary intervals for CA and CC indices

Below is some code to estimate bootstrap confidence intervals and Bayesian credible intervals for CA and CC indices as described in the main text. To illustrate the procedures, we simulate ten factor model parameters and responses from 500 individuals.

## Data Simulation

Ten factor loadings were drawn from a uniform distribution with .5 and .9 as the limits, residual variances were the compliments of the factor loading so that the item total variance for each item was 1, and the item intercepts were all set to zero. Latent variables scores for individuals were drawn from a standardized normal distribution. We will estimate the classification rate, sensitivity, specificity, and classification consistency at the cutpoint of 1SD for the observed scores and latent variable scores.

#load packages needed
library(psych)
library(lavaan)
library(mvtnorm)
library(R2jags)

#TRUE VALUES
set.seed(201003)
k1=runif(10,.5,.9) #factor loadings
k2=1-k1^2 #residual variances
k3=0 #intercepts

#see factor model parameters
dat=data.frame(k1,k2,k3)
dat

## k1 k2 k3
## 1 0.8094423 0.3448032 0
## 2 0.5394930 0.7089473 0
## 3 0.7119611 0.4931114 0
## 4 0.7218067 0.4789951 0
## 5 0.8139162 0.3375404 0
## 6 0.7188974 0.4831866 0
## 7 0.8834345 0.2195434 0
## 8 0.7056338 0.5020809 0
## 9 0.6011867 0.6385746 0
## 10 0.5506165 0.6968214 0

#simulate 500 cases with helper function
dat2=cont_sim(rnorm(500),dat)
quantile(rowSums(dat2),prob=.84) #observed cutpoint at 1sd, which is ~ 8

## 84%
## 7.94496

## Estimating the CA and CC indices

First, we fit a unidimensional factor model to the data, save the factor model parameters, and use the helper functions provided at the end of the document to estimate the model implied relations of the summed score and the CA and CC indices. Note that the helper functions provide other useful estimates: the sum of the loadings, intercepts, and residual variances, the estimates of the four quadrants (botleft, topleft, botright,topright) needed for the CA indices, and the two quadrants (upcorner, downcorner) needed to estimate the CC indices.

mod0=paste0('f1=~',paste0('V',1:10,collapse='+')) #write model
fit0=cfa(mod0,data=dat2,std.lv=TRUE,meanstructure=TRUE) #fit model
summary(fit0) #see estimates

## lavaan 0.6-9 ended normally after 21 iterations
##
## Estimator ML
## Optimization method NLMINB
## Number of model parameters 30
##
## Number of observations 500
##
## Model Test User Model:
##
## Test statistic 16.122
## Degrees of freedom 35
## P-value (Chi-square) 0.997
##
## Parameter Estimates:
##
## Standard errors Standard
## Information Expected
## Information saturated (h1) model Structured
##
## Latent Variables:
## Estimate Std.Err z-value P(>|z|)
## f1 =~
## V1 0.787 0.036 21.554 0.000
## V2 0.529 0.044 11.986 0.000
## V3 0.681 0.039 17.261 0.000
## V4 0.760 0.040 19.227 0.000
## V5 0.807 0.038 21.488 0.000
## V6 0.737 0.041 18.143 0.000
## V7 0.924 0.037 25.219 0.000
## V8 0.760 0.040 18.820 0.000
## V9 0.617 0.042 14.846 0.000
## V10 0.513 0.040 12.808 0.000
##
## Intercepts:
## Estimate Std.Err z-value P(>|z|)
## .V1 -0.027 0.043 -0.614 0.539
## .V2 0.052 0.046 1.123 0.261
## .V3 0.003 0.044 0.061 0.951
## .V4 0.030 0.045 0.668 0.504
## .V5 0.058 0.045 1.294 0.196
## .V6 -0.001 0.046 -0.030 0.976
## .V7 0.018 0.046 0.382 0.702
## .V8 -0.002 0.046 -0.046 0.963
## .V9 0.007 0.045 0.152 0.879
## .V10 0.027 0.042 0.651 0.515
## f1 0.000
##
## Variances:
## Estimate Std.Err z-value P(>|z|)
## .V1 0.326 0.024 13.559 0.000
## .V2 0.773 0.050 15.389 0.000
## .V3 0.504 0.034 14.732 0.000
## .V4 0.454 0.032 14.309 0.000
## .V5 0.347 0.026 13.586 0.000
## .V6 0.510 0.035 14.559 0.000
## .V7 0.217 0.019 11.135 0.000
## .V8 0.485 0.034 14.408 0.000
## .V9 0.624 0.041 15.096 0.000
## .V10 0.621 0.041 15.317 0.000
## f1 1.000

dat0=matrix(coef(fit0),ncol=3,byrow=F) #factor model params to matrix
dat0

## [,1] [,2] [,3]
## [1,] 0.7867072 0.3257771 -0.026702
## [2,] 0.5286676 0.7733868 0.051548
## [3,] 0.6808113 0.5043765 0.002696
## [4,] 0.7603269 0.4535513 0.030342
## [5,] 0.8073196 0.3471301 0.057852
## [6,] 0.7366695 0.5100222 -0.001398
## [7,] 0.9244173 0.2166193 0.017690
## [8,] 0.7599689 0.4849380 -0.002132
## [9,] 0.6169319 0.6235326 0.006820
## [10,] 0.5125276 0.6211564 0.027376

#helper functions: cont_cc and sesp
tv1=cont_cc(m_theta=0,v_theta=1,matpar=dat) #get implied moments
tv1 #sd_local is error variance at each level of theta

## mean_x var_x sd_x sd_local cor_x sum_lam sum_epi sum_int
## 0.000 54.696 7.396 2.214 0.954 7.056 4.904 0.000

tv2=round(sesp(tv1,cutobs=8,cut_theta=1),3) #get CA and CC indices
tv2

## cr sens spec cons topleft botleft topright
## 0.942 0.757 0.977 0.925 0.020 0.822 0.120
## botright upcorner downcorner
## 0.039 0.102 0.823

## Bootstrap confidence intervals for CA and CC indices

The procedure to estimate bootstrap confidence intervals is the following:

1. The dataset is resampled with replacement
2. A factor model is fit to the resampled dataset and factor model parameters are saved
3. Model-implied moments of the summed score are calculated from the factor model parameters
4. Bivariate normal distributions to estimate CA and CC indices are calculated from the implied moments, and estimates are saved.
5. Steps 1-4 are repeated many times (e.g., 500x)
6. Summarize empirical distribution of the indices using distribution percentiles to form an equal-tail confidence interval or build it iteratively using HPD intervals.

boot1=list(NULL) #list to save results

for(i in 1:500){ #500 bootstrap samples
 ss=sample(1:nrow(dat2),nrow(dat2),replace = TRUE)
 dat3=dat2[ss,]

 #fit model
 fit1=cfa(mod0,data=dat3,std.lv=TRUE,meanstructure=TRUE)

 #save coefficients
 boot1[[i]]=coef(fit1)
}

#turn each coefficient set to a matrix
nl=function(x){
 matrix(x,ncol=3,nrow=10,byrow=F)
}
b3=lapply(boot1,nl)

#get implied moments in each bootstrap sample
b4=lapply(b3,function(x) cont_cc(m_theta=0,v_theta=1,matpar=x))

#get CA and CC indices at each boostrap sample
b5=lapply(b4,sesp,cutobs=8,cut_theta=1)
b6=do.call(rbind,b5)

#equal tail confidence interval
aa1=round(summary(as.mcmc(b6),quantiles=c(.025,.5,.975))[[2]],3)
aa1

## 2.5% 50% 97.5%
## cr 0.936 0.943 0.946
## sens 0.684 0.784 0.858
## spec 0.957 0.973 0.985
## cons 0.916 0.924 0.931
## topleft 0.013 0.023 0.036
## botleft 0.806 0.818 0.829
## topright 0.109 0.124 0.136
## botright 0.023 0.034 0.050
## upcorner 0.086 0.109 0.130
## downcorner 0.787 0.814 0.844

#HPD confidence interval
aa2=round(HPDinterval(as.mcmc(b6),prob=.95),3)
aa2

## lower upper
## cr 0.936 0.946
## sens 0.695 0.866
## spec 0.959 0.986
## cons 0.916 0.931
## topleft 0.012 0.034
## botleft 0.807 0.830
## topright 0.110 0.137
## botright 0.021 0.048
## upcorner 0.087 0.131
## downcorner 0.785 0.841
## attr(,"Probability")
## [1] 0.95

## Bayesian credible interval for CA and CC indices

The procedure to estimate Bayesian credible intervals is the following:

1. Estimate a factor model using MCMC and save draws from the posterior distribution of the factor loadings, item intercepts, and residual variances.
2. Model-implied moments of the summed score are calculated for each set of draws from the factor model parameter posterior distributions.
3. Bivariate normal distributions to estimate CA and CC indices are calculated from the implied moments, and estimates are saved.
4. Summarize posterior distribution of the indices using percentiles to form an equal-tail credible interval or build it iteratively using HPD credible intervals.

bcefa=function() {

# Specify the factor analysis measurement model for the observables

 for (i in 1:n){
 for(j in 1:J){
 mu[i,j] <- tau[j] + ksi[i]*lambda[j] # model implied response
 x[i,j] ~ dnorm(mu[i,j], inv.psi[j]) # distribution per response
 }
 }


# Specify the (prior) distribution for the latent variables

 for (i in 1:n){
 ksi[i] ~ dnorm(kappa, inv.phi) # distribution for the latent var
 }

# Specify priors for the latent variables

 kappa <- 0 # Mean of factor 1
 inv.phi <- 1 # Precision of factor 1

# Specify the prior distribution for the factor model parameters

 for(j in 1:J){
 tau[j] ~ dnorm(3, .1) # Intercepts for item response
 inv.psi[j] ~ dgamma(5, 10) # Precisions for item response
 psi[j] <- 1/inv.psi[j] # Variances for item response
 }

 for (j in 1:J){
 lambda[j] ~ dnorm(1, .1) # prior distribution for loadings
 }

}

jagsdat=list(x=dat2,n=nrow(dat2),J=ncol(dat2)) #data to JAGS
bpar <- c("tau", "lambda", "psi") #parameters to save

#fit model
jagcfa <- jags(data = jagsdat,
 parameters.to.save = bpar, n.chains=3,
 n.iter = 5000, n.burnin = 1000, model.file = bcefa)

# save draws and drop deviance from the model
l1=do.call(rbind,as.mcmc(jagcfa))[,-1]

#get matrix of factor model parameters per draw
l2=split(l1,1:nrow(l1))
nl=function(x){
 matrix(x,ncol=3,nrow=10,byrow=F)
}
l3=lapply(l2,nl)

#get implied moments per each set of mcmc draws
p1=lapply(l3,function(x) cont_cc(m_theta=0,v_theta=1,matpar=x))

#get CA and CC indices per set of draws
p2=lapply(p1,sesp,cutobs=8,cut_theta=1)
p3=do.call(rbind,p2)

#equal tail credible intervals
aa3=round(summary(as.mcmc(p3),quantiles=c(.025,.5,.975))[[2]],3)
aa3

## 2.5% 50% 97.5%
## cr 0.936 0.941 0.944
## sens 0.700 0.788 0.866
## spec 0.952 0.970 0.982
## cons 0.913 0.920 0.928
## topleft 0.015 0.025 0.040
## botleft 0.801 0.816 0.826
## topright 0.111 0.125 0.137
## botright 0.021 0.034 0.048
## upcorner 0.090 0.110 0.134
## downcorner 0.779 0.810 0.837

#HPD credible intervals
aa4=round(HPDinterval(as.mcmc(p3),prob=.95),3)
aa4

## lower upper
## cr 0.937 0.945
## sens 0.699 0.865
## spec 0.954 0.983
## cons 0.913 0.927
## topleft 0.014 0.038
## botleft 0.803 0.827
## topright 0.111 0.137
## botright 0.021 0.048
## upcorner 0.089 0.134
## downcorner 0.782 0.838
## attr(,"Probability")
## [1] 0.95

## Helper functions

Below are three helper functions used in this appendix

1. cont_sim(): Function to simulate data
2. cont_cc(): Function to estimate implied summed score moments from the factor parameters
3. sesp(): Function that takes the implied summed score moments and the cutscores to estimate the classification rate, sensitivity, specificity, and classification consistency

#function to simulate data from factor model params and latent var scores
cont_sim=function(theta,matpar){
 Nr=length(theta)
 u=matrix(NA,ncol=nrow(matpar),nrow=Nr)
 for(i in 1:Nr){
 u[i,]=round(matpar[,1]*theta[i]+rnorm(nrow(matpar),0,sqrt(matpar[,2]))+matpar[,3],3)
 }
 i2=u
 colnames(i2)=c(paste0('V',1:nrow(matpar)))
 i2
}

#function to get model-implied moments for summed scores
cont_cc=function(m_theta,v_theta,matpar){

 mean_x=sum(matpar[,3])+sum(matpar[,1])*m_theta
 var_x=sum(matpar[,1])^2*v_theta+sum(matpar[,2])
 sd_x=sqrt(var_x)
 sd_local=sqrt(sum(matpar[,2]))
 cor_x=sum(matpar[,1])/sqrt(sum(matpar[,1])^2+sum(matpar[,2]))
 sumpars=colSums(matpar)


 aa=round(c(mean_x,var_x,sd_x,sd_local,cor_x,sumpars),3)
 names(aa)=c('mean_x','var_x','sd_x','sd_local','cor_x','sum_lam','sum_epi','sum_int')
 aa
}

#function to estimate CA and CC indices as described in the text
sesp=function(x,cut_theta=NULL,cutobs){
 if(is.null(cut_theta) & !is.null(cutobs)){
 cut_theta=(cutobs-x['sum_int'])/x['sum_lam']
 }
 if(is.null(cutobs) & !is.null(cut_theta)){
 cutobs=x['sum_int']+x['sum_lam']*cut_theta
 }

 S=matrix(c(1,x['cor_x']*x['sd_x'],x['cor_x']*x['sd_x'],x['var_x']),nrow=2,byrow=T)
 topleft=pmvnorm(lower=c(-Inf,cutobs),upper=c(cut_theta,Inf),mean=c(0,x['mean_x']),sigma=S)
 botleft=pmvnorm(lower=c(-Inf,-Inf),upper=c(cut_theta,cutobs),mean=c(0,x['mean_x']),sigma=S)
 topright=pmvnorm(lower=c(cut_theta,cutobs),upper=c(Inf,Inf),mean=c(0,x['mean_x']),sigma=S)
 botright=pmvnorm(lower=c(cut_theta,-Inf),upper=c(Inf,cutobs),mean=c(0,x['mean_x']),sigma=S)
 a1=matrix(c(topleft,botleft,topright,botright),nrow=1)
 colnames(a1)=c('topleft','botleft','topright','botright')

 classrate=topright+botleft
 sensitivity=topright/(topright+botright)
 specificity=botleft/(botleft+topleft)

 S2=matrix(c(x['var_x'],(x['cor_x']*x['sd_x'])^2,(x['cor_x']*x['sd_x'])^2,x['var_x']),nrow=2,byrow=T)
 downcorner=pmvnorm(lower=c(-Inf,-Inf),upper=c(cutobs,cutobs),mean=c(x['mean_x'],x['mean_x']),sigma=S2)
 upcorner=pmvnorm(lower=c(cutobs,cutobs),upper=c(Inf,Inf),mean=c(x['mean_x'],x['mean_x']),sigma=S2)
 a11=matrix(c(upcorner,downcorner),nrow=1)
 colnames(a11)=c('upcorner','downcorner')

 consistency=upcorner+downcorner


 a2=c(classrate,sensitivity,specificity,consistency,a1,a11)
 names(a2)=c('cr','sens','spec','cons',colnames(a1),colnames(a11))
 return(a2)
}
